# Soybean *GmSNF4* Confers Salt–Alkali Stress Tolerance in Transgenic Plants

**DOI:** 10.3390/plants14142218

**Published:** 2025-07-17

**Authors:** Nan Ye, Jia-Shen Bian, Bai-Hui Zhou, Ling-Tao Yong, Ting Yang, Nan Wang, Yuan-Yuan Dong, Wei-Can Liu, Fa-Wei Wang, Hai-Yan Lv, Xiao-Wei Li

**Affiliations:** 1 College of Life Sciences, Jilin Agricultural University, Changchun 130118, China; 18943117963@163.com (N.Y.); 13504473369@163.com (J.-S.B.); 15754366701@163.com (B.-H.Z.); 18844065895@163.com (L.-T.Y.); yangt686@163.com (T.Y.); wangnanlunwen@126.com (N.W.); yydong@aliyun.com (Y.-Y.D.); liuweican602@163.com (W.-C.L.); fw-1980@163.com (F.-W.W.); 2 Institute of Crop Germplasm Resources, Jilin Academy of Agricultural Sciences (Northeast Agricultural Research Center of China), Gongzhuling 136100, China; 3 College of Horticulture, Jilin Agricultural University, Changchun 130118, China

**Keywords:** *GmSNF4*, soybean, salt–alkali stress, transgenic plants

## Abstract

In order to mitigate the reduction in soybean yield caused by soil salinization, a soybean gene, *GmSNF4*, which promotes plant tolerance to salt–alkali stress, was identified in this study. The STRING database was used to predict the interaction between GmSNF4 and GmPKS4. The *GmPKS4* gene was experimentally shown to be involved in salt–alkali stress tolerance. Firstly, the yeast two-hybrid technique and bimolecular fluorescence complementation (BiFC) technique were used to confirm the interaction between GmSNF4 and GmPKS4: the AMPK-CBM-CBS1 conserved domain was thereby determined to be the region of the GmSNF4 protein involved in the interaction. Secondly, the *GmSNF4* gene was induced by salt–alkali stress according to qRT-PCR analysis, and the GmSNF4 protein was localized in the nucleus and cytoplasm. Finally, analysis of *GmSNF4*’s role in resistance to salt–alkali stress in transgenic soybean plants showed that transgenic lines had better phenotypic, physiological, and stress-related gene expression than non-transgenic soybeans. Thus, *GmSNF4* may play a significant role in plant salt–alkali stress tolerance.

## 1. Introduction

Soybean (*Glycine max*) is the main variety of feed and industrial crop in China due to its rich protein and oil contents. However, soybean production is significantly affected by various adverse stresses, among which salt–alkali stress is particularly noteworthy [1]. Salt–alkali soils are widely distributed in many regions of China, including the Northeast Plain, the North China Plain, and some coastal areas [2]. These soils are characterized by high salinity and alkalinity, which create harsh conditions for plant growth. For soybeans, salt–alkali stress can lead to osmotic stress, ion toxicity, and oxidative damage. It disrupts the water balance within the plant, inhibits nutrient uptake, and impairs photosynthesis and respiration processes, ultimately resulting in reduced growth, lower yields, and even plant death [3]. Therefore, one effective method for improving soybean yield under such conditions is the application of salt–alkali stress response genes in molecular breeding [1].

Salt–alkali stress, drought, high temperature, and other environmental stresses rapidly alter redox homeostasis, which causes serious oxidative damage to plant cells [4]. The plant antioxidant system regulates the accumulation of osmotic-regulating substances, hormone content, and the expression of related genes, among other factors [4]. Improving this system is therefore an important method for improving salt–alkali stress tolerance, and effectively reducing stress injury in plants. The core stress response signal involves protein kinase SnRK1 (sucrose non-fermenting-1-related protein kinase 1), which is homologous to yeast sucrose non-fermenting 1 (SNF1) and mammalian adenosine 5′-monophosphate (AMP)-activated protein kinase (AMPK) [5].

SNF1-related protein kinase 1 (SnRK1) is the plant ortholog of SNF1 in budding yeast and AMPK in mammals. These kinases belong to a highly conserved eukaryotic protein kinase family of metabolic sensors and are activated in response to declining energy levels. Upon activation, SNF1/AMPK/SnRK1 kinases trigger extensive transcriptional and metabolic reprogramming, restoring energy homeostasis and promoting tolerance to adverse conditions, partly through the induction of catabolic processes and generally inhibiting anabolism [6]. SnRK1 exists as a heterotrimeric complex consisting of an α-catalytic subunit, a β-regulatory subunit, and a γ or βγ-regulatory subunit. Due to the existence of multiple isoforms for each subunit, various isoenzymes are generated. Additionally, two atypical subunits, β3 and βγ, have been found in plants [6]. The first evidence for the functional conservation of plant orthologs and the regulation of energy metabolism by SnRK1 came from the complementation of the yeast SNF1 mutant with a SNF1-related cDNA from rye. This complementation restored the utilization of non-fermentable carbon sources such as ethanol and glycerol, indicating that the rye SNF1-related cDNA can substitute for SNF1 in the sugar signaling pathway. Similar results were obtained using SnRK1 from other plant species, such as tobacco, potato, and *Arabidopsis*, in yeast complementation assays [7]. Existing research shows that SnRK1 is involved in the regulation of various physiological and biochemical processes in plants and links stress and metabolism [8]. The overexpression of the catalytic subunit of SnRK1 causes delays in *Arabidopsis* flowering and defects in the formation of horned fruits and cotyledons, and this phenotype can be alleviated by the fus3 mutant [5]. SnRK1 and FUS3 (Fused in Sarcoma 3) interact and regulate the stability of the FUS3 protein in plants, which is also regulated by ABA (abscisic acid) [9]. Studies have shown that the activity of SnRK1 is inhibited by PP2Cs (protein phosphatases type 2C), which are a family of proteins in the ABA signaling pathway and inhibit SnRK2 activity [10,11]. In addition, the overexpression of SnRK1 can increase the sensitivity of *Arabidopsis* to glucose and ABA. The addition of glucose during ABA treatment enhances the sensitivity of SnRK1-overexpressing plants to ABA, suggesting that plant stress signaling is mediated through energy sensing [12].

The gene expression profile of soybean was analyzed using high-throughput Illumina sequencing combined with bioinformatics. It was found that 1897 and 1731 genes were significantly upregulated in leaves and roots, respectively, after salt–alkali stress treatment [12]. Among these genes, the expression of *GmPKS4* was significantly upregulated in both roots and leaves, indicating that this gene may be closely related to salt–alkali stress [13]. It can be inferred that *GmPKS4* transgenic *Arabidopsis thaliana* and composite plants with soybean hairy roots exhibit tolerance to salt and salt–alkali stress [14,15]. Thus, *GmPKS4* is an important candidate gene resource for salt–alkali tolerance in soybean [14]. Using the STRING database, it was predicted that a soybean GmSNF4 protein interacts with GmPKS4. It was found that SNF4 is a β γ subunit of the SnRK1 heterotrimer complex. Meanwhile, bioinformatics analysis showed that GmSNF4 is a CUB domain-containing protein (CDCP) containing the cystathionine β synthase (CBS) domain. According to research, the CBSDUF protein belongs to the CDCP superfamily, which contains a domain of unknown function (DUF21) and an N-terminus adjacent to two intracellular CBS domains [16]. In addition, *GmCBSDUF3* transgenic *Arabidopsis* was subjected to phenotypic analysis under NaCl, PEG, and ABA stress treatment. The overexpression of *GmCBSDUF3* could enhance tolerance to drought and salt in *Arabidopsis* [16]. CDCP may also play an important role in the stress response and tolerance to salt stress, heavy metals, and oxidation in plants. Therefore, we speculated that *GmSNF4* is also involved in salt–alkali stress resistance in plants. In this study, the soybean *GmSNF4* gene was identified and characterized. The interaction between GmSNF4 and GmPKS4 was verified using yeast two-hybrid and BiFC technology. GmSNF4 was found to be located in the cytoplasm and nucleus. The expression pattern of *GmSNF4* were studied, and a salt–alkali resistance function was initially identified. The phenotype, stomata, and ROS content of *GmSNF4*-overexpressing (*GmSNF4*-OE) soybeans indicate that *GmSNF4*-OE is tolerant to salt–alkali stress, suggesting that *GmSNF4* is involved in the salt–alkali resistance of plants.

## 2. Results

### 2.1. Identification of Interaction Between GmSNF4 and GmPKS4

SNF4 is the beta-gamma subunit that forms the SnRK1 heterotrimeric complex, which is unique to green plants. It contains a carbohydrate-binding module (CBM) at the N-terminus (usually present in certain beta subunits) and a cystathionine beta-synthase domain (CBS) commonly found at the C-terminus of the gamma subunit (Figure S1) [17]. The CBM of SNF1/AMPK/SnRK1 belongs to the CBM20 family of CBMs. In mammals, it has been proposed that the glycogen-binding domain (GBD) acts as a regulatory domain for inhibiting AMPK activity, as this domain binds to glycogen *in vitro* and acts as a sensor for glycogen, which serves as a stored carbon source [17].

**Figure 1:**
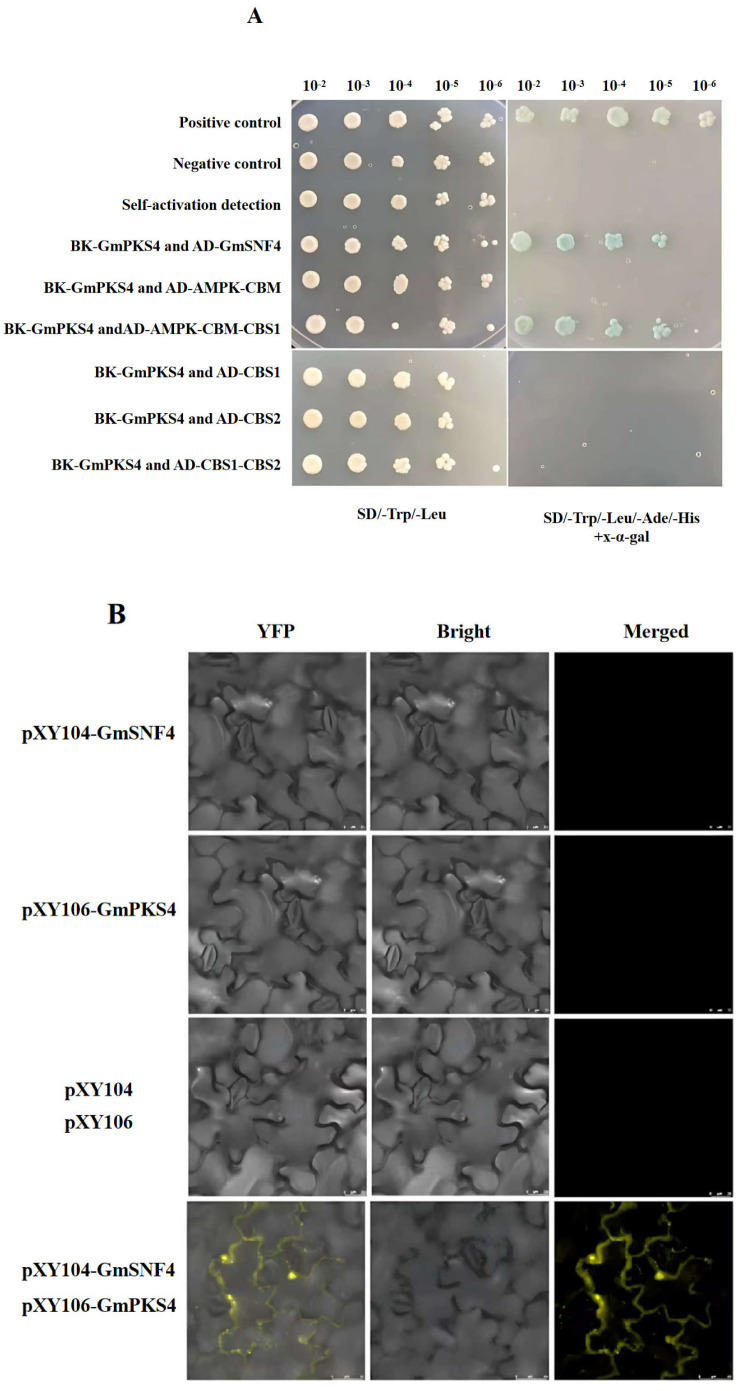
GmSNF4 interacts with GmPKS4. (**A**) Yeast two-hybrid assays: The GmSNF4 and GmPKS4 genes were cloned into AD or BK vectors. The resulting plasmids or control vectors were co-transformed into Y2HGold yeast cells. The transformed clones were grown on selective media and medium containing X-α-gal. Blue colonies indicate interactions. Co-transformations with pGADT7-T+pGBKT7-53 and pGADT7-T+pGBKT7-Lam were used as positive and negative controls, respectively. (**B**) BiFC assay: Clone the *GmSNF4* and *GmPKS4* genes into the vectors pXY104 and pXY106 respectively, and co-transform the constructed recombinant plasmids into tobacco leaf mesophyll cells for expression for 48 h. YFP signals were observed under a confocal microscope.

In order to verify the interaction between soybean proteins GmSNF4 and GmPKS4, we used pGBKT7-53+pGADT7-T as a positive control and pGBKT7-lam+pGADT7-T as a negative control. The constructed pGBKT7-GmPKS4+pGADT7-GmSNF4 was co-transformed into the Y2HGold yeast strain (Figures S2 and S3). Perform yeast transformation and plating according to Table S2. The clones grew normally on SD/-Trp/-Leu medium, indicating that the positive, negative, and hybrid groups were successfully transformed into competent cells. However, only the negative group could not grow on SD/-Trp/-Leu/-Ade/-His medium, indicating that the *ADE2* and *HIS3* reporter genes were activated. Both the positive and hybrid groups showed blue color on SD/-Trp/-Leu/-Ade/-His+X-α-gal substrate display medium, indicating that the *MEL1* gene was activated and that the two proteins GmPKS4 and GmSNF4 could interact well. The interaction region of the GmSNF4 protein was identified as AMPK-CBM-CBS1 (Figure 1A).

To further confirm the interaction between GmSNF4 and GmPKS4, pXY104-GmSNF4 and pXY106-GmPKS4 were constructed and transformed into tobacco mesophyll cells via *Agrobacterium tumefaciens* transformation (Figures S4 and S5) [18,19]. Yellow fluorescence signals were not observed in tobacco mesophyll cells transformed with pXY104, pXY106, pXY104-GmSNF4, or pXY106-GmPKS4 alone, nor in cells co-transformed with pXY104 and pXY106 under a laser confocal scanning microscope. However, when pXY104-GmSNF4 and pXY106-GmPKS4 were co-transformed into tobacco mesophyll cells, a specific yellow fluorescent signal was observed under the microscope, indicating the interaction between the GmSNF4 and GmPKS4 proteins (Figure 1B).

### 2.2. Analysis of GmSNF4 Gene Expression and Subcellular Localization of GmSNF4 Protein

**Figure 2:**
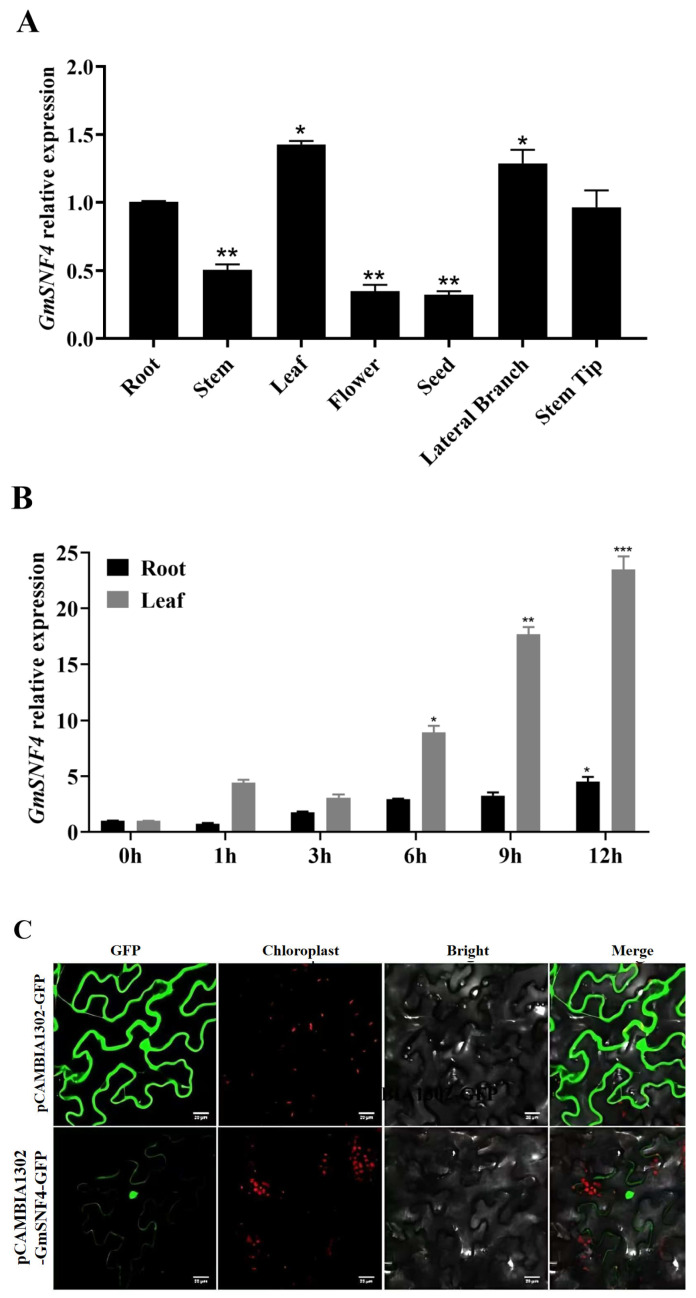
The relative expression of the soybean *GmSNF4* gene and the subcellular localization of the GmSNF4 protein under salt–alkali stress. (**A**) Relative expression levels of the *GmSNF4* gene in different tissues of transgenic soybeans, with roots as the control group. (**B**) Using the gene expression level at 0 h as the control group, the relative expression levels of the *GmSNF4* gene in the roots and leaves of soybeans under salt–alkali stress at 0, 1, 3, 6, 9, and 12 h; the salt–alkali concentrations were 70 mM NaCl and 50 mM NaHCO_3_. The data represent the means and standard deviations of three repeats (*n* = 3). Significant differences were determined with unpaired two-sided Student’s *t*-tests (*, *p* < 0.05; **, *p* < 0.01; ***, *p* < 0.001). (**C**) The subcellular localization of the soybean GmSNF4 protein. GFP: green fluorescent protein; Bright: bright field; Merged: superposition field; scale: 50 μm.

Quantitative analysis was conducted on different tissues of soybean, andthe expression of the *GmSNF4* gene in soybean tissues such as roots, stems, leaves, flowers, seeds, lateral branches, and stem tips was detected by quantitative real-time PCR (qRT-PCR). The results showed that the *GmSNF4* gene was expressed in all tissues, with the highest relative expression level in leaves compared to roots. This suggests that the gene plays a role in plant growth and development (Figure 2A). Soybean seedlings were subjected to salt–alkali stress (70 mM NaCl and 50 mM NaHCO_3_) for 12 h, and the expression of the *GmSNF4* gene in the roots and leaves was measured via qRT-PCR. The results showed that the expression of the *GmSNF4* gene in soybean roots and leaves increased significantly under salt–alkali stress compared to the control group, indicating that the *GmSNF4* gene is closely related to salt–alkali stress (Figure 2B).

The function of a protein is closely related to its subcellular localization. To predict the localization of the GmSNF4 protein, we used the PSORT (http://www.genscript.com/psort/wolf_psort.html/, accessed on 11 June 2020) and TargetP (https://services.healthtech.dtu.dk/services/TargetP-2.0/) subcellular localization methods. These methods predicted that GmSNF4 is localized in the cytoplasm or nucleus. To verify the protein’s specific localization, we constructed a GFP fusion expression vector, pCAMBIA1302-GmSNF4-GFP (Figure S6), and transiently expressed it in tobacco mesophyll cells using *Agrobacterium tumefaciens*. Fluorescence signals were observed under a laser confocal microscope with an excitation wavelength of 488 nm and an emission wavelength of 507 nm. The results showed that GmSNF4-GFP signals accumulated in both the nucleus and cytoplasm (Figure 2C).

### 2.3. The GmSNF4 Gene Enhanced the Salt–Alkali Tolerance of Soybean

**Figure 3:**
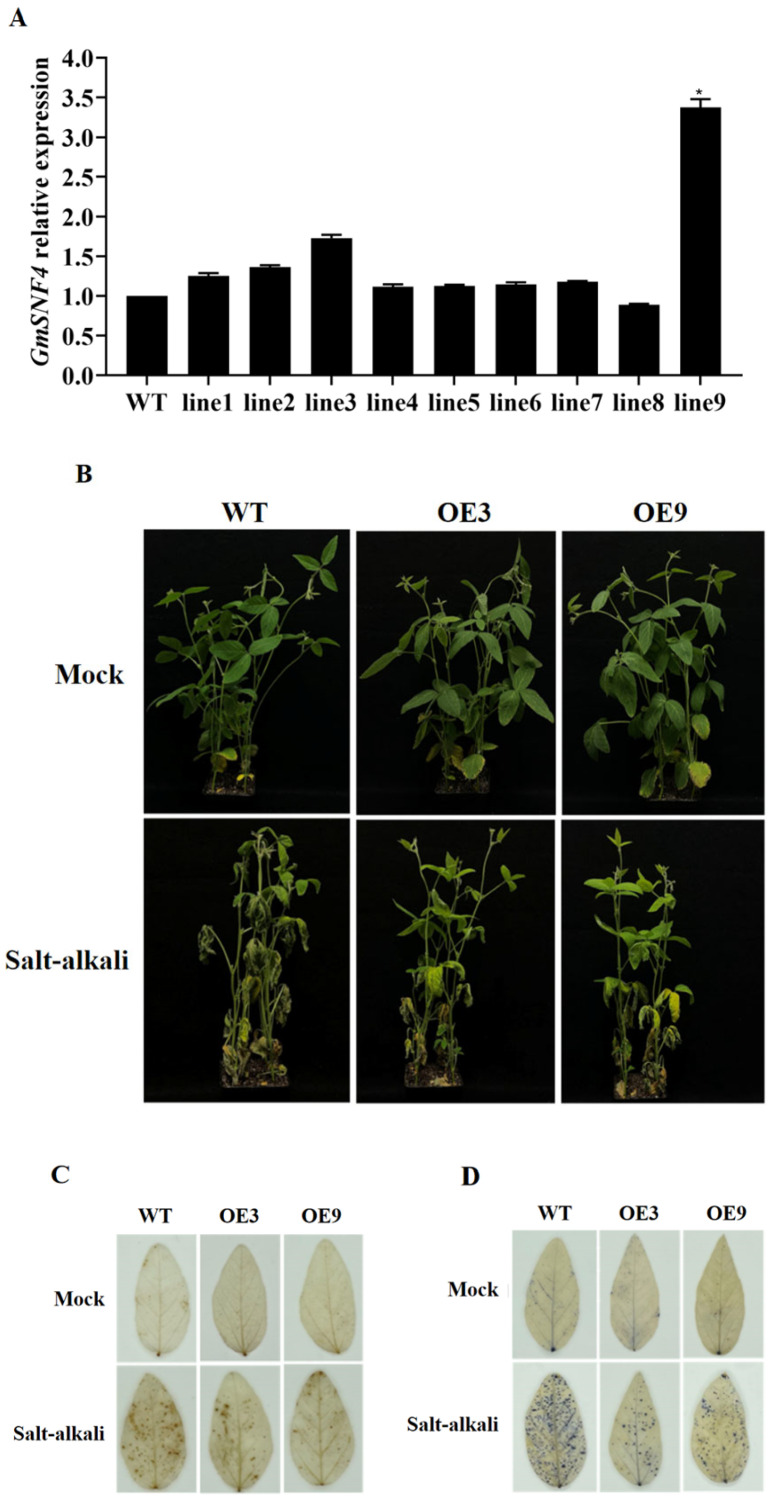
Response of transgenic soybean plants with the *GmSNF4* gene to salt–alkali stress. (**A**) Analysis of the relative expression level of the *GmSNF4* gene in the T2 transgenic soybean line, with the WT gene expression level as the control group. (**B**) The phenotype of soybeans that were transformed with the *GmSNF4* gene under salt–alkali stress. (**C**) DAB staining of soybean leaves under salt–alkali stress. (**D**) NBT staining of soybean leaves under salt–alkali stress. Significant differences were determined using unpaired two-sided Student’s *t*-tests (*, *p* < 0.05).

In order to characterize the function of *GmSNF4*, five T1 transgenic soybean lines were cultivated via *Agrobacterium*-mediated transformation (Figures S9 and S10) [20]. The *GmSNF4* level in each T2 generation line was examined using qRT-PCR. Of these nine lines, OE3 and OE9, with relatively high *GmSNF4* expression levels, were analyzed further (Figure 3A). Specifically, T3 generation lines were produced from these lines, after which the seeds were collected for use as experimental materials, with non-transferred soybean (WT) plants serving as controls.

Four-week-old WT, OE3, and OE9 seedlings were subjected to salt–alkali stress (90 mM NaCl + 60 mM NaHCO_3_) for 10 days (see Section 4.3 for details) to further explore the response of the *GmSNF4* gene to salt–alkali stress. Under normal conditions without salt–alkali stress treatment, the phenotypic growth of wild-type plants was similar to that of transgenic plants. After 10 days of salt–alkali stress treatment, the bottom leaves of wild-type plants turned yellow and exhibited wilting. In contrast, transgenic plants with the *GmSNF4* gene showed less damage (Figure 3B).

**Figure 4:**
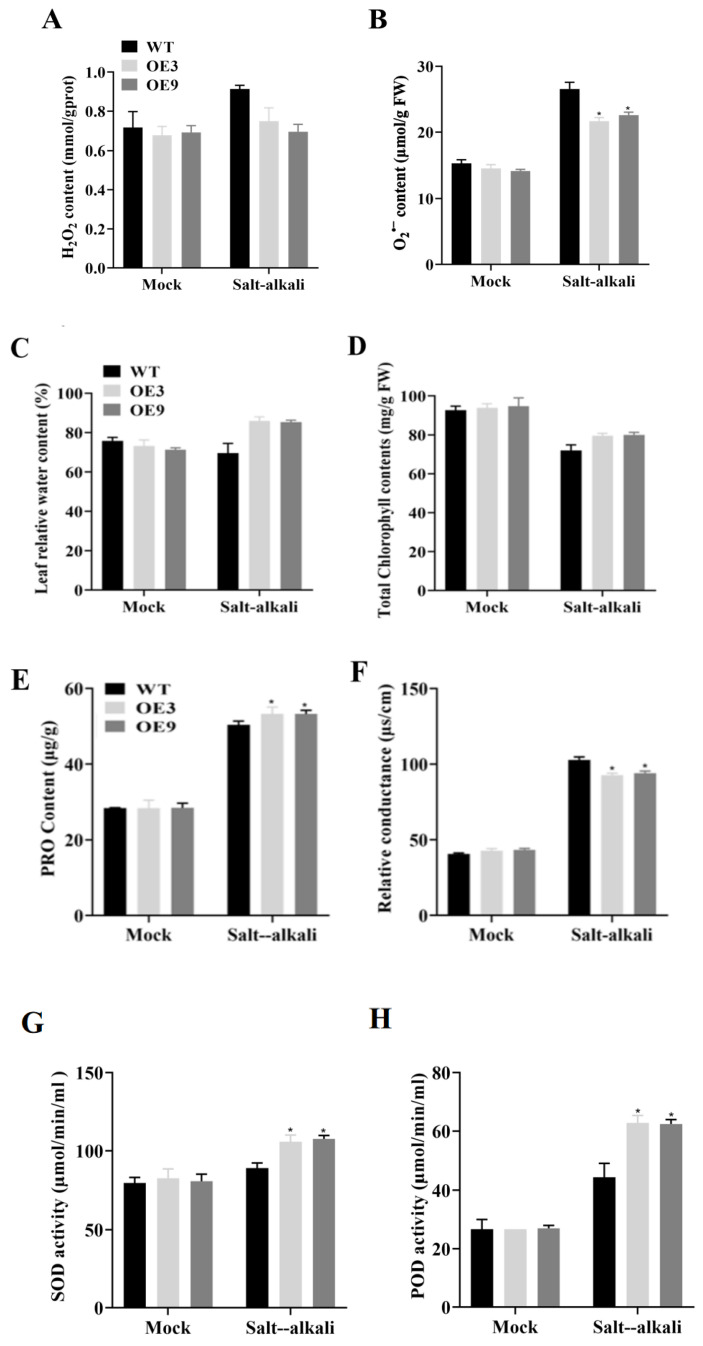

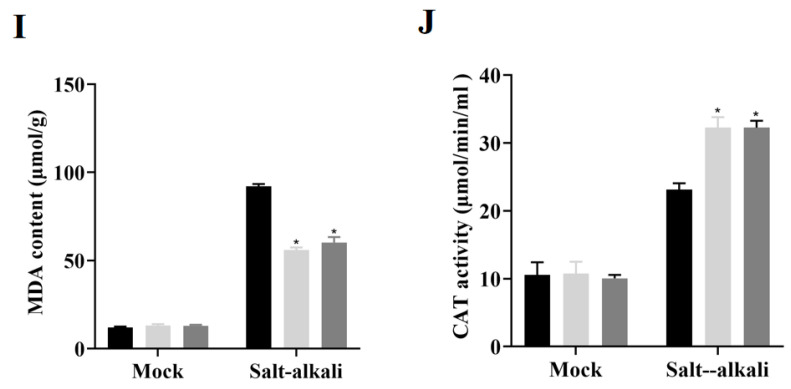
Physiological index analysis of transgenic soybean leaves with *GmSNF4* gene under salt–alkali stress. (**A**) Hydrogen peroxide accumulation in GmSNF4 gene soybean. (**B**) Accumulation of O_2_^•−^ in transgenic soybean. (**C**) Relative leaf water content of *GmSNF4* transgenic soybean leaves. (**D**) Chlorophyll content of transgenic soybean leaves with *GmSNF4* gene. (**E**) PRO activity of *GmSNF4* transgenic soybean. (**F**) Relative conductivity of transgenic soybean leaves with *GmSNF4* gene. (**G**) SOD activity of *GmSNF4* transgenic soybean. (**H**) POD activity of *GmSNF4* transgenic soybean. (**I**) MDA content of *GmSNF4* transgenic soybean. (**J**) CAT activity of *GmSNF4* transgenic soybean. Significant differences were determined using unpaired two-sided Student’s *t*-tests (*, *p* < 0.05).

The cell viability of soybean leaves under salt–alkali stress was measured via DAB (3,3-diaminobenzidine) and NBT (nitroblue tetrazolium) staining (Figure 3C,D) to directly determine the degree of leaf damage [21,22]. The results showed that the staining intensity of transgenic plants was significantly lower than that of wild-type plants under salt–alkali stress, indicating that transgenic plants were less damaged than wild-type plants. This finding was consistent with the determinations of hydrogen peroxide and superoxide anion content (Figure 4A,B). Additionally, the relative water content and chlorophyll content of leaves were measured. The results indicated that the phenotype of transgenic plants was consistent with that of wild-type plants under normal conditions, but transgenic plants exhibited significant growth advantages under salt–alkali stress (Figure 4C,D). Oxidative damage caused by salt–alkali stress can lead to damage to the physiological system, resulting in the leakage of electrolytes and excessive accumulation of MDA (malondialdehyde), the end product of membrane lipid peroxidation. Under salt–alkali stress, the MDA content and electrical conductivity of transgenic plants were lower than those of wild-type plants (Figure 4F,I) [23,24]. Moreover, the contents of proline (PRO), superoxide dismutase (SOD), peroxidase (POD), and catalase (CAT) in transgenic plants were higher than those in wild-type plants (Figure 4E,G,H,J). These results suggest that the membrane lipids of transgenic soybean plants were less damaged, and the *GmSNF4* gene could enhance plant tolerance by protecting them from the adverse effects of oxidative stress and osmotic stress.

### 2.4. Analysis of Relative Expression Levels of Stress-Related Genes in GmSNF4-Transgenic Soybean

**Figure 5:**
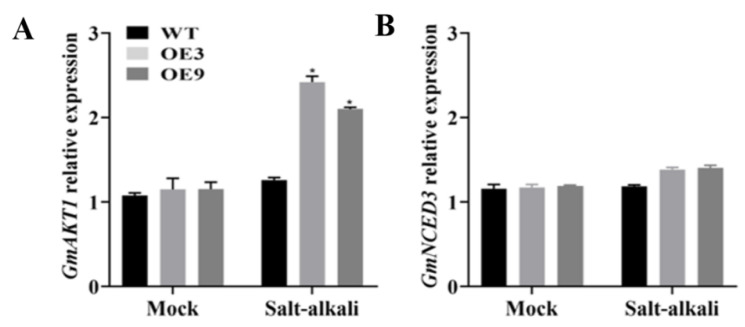

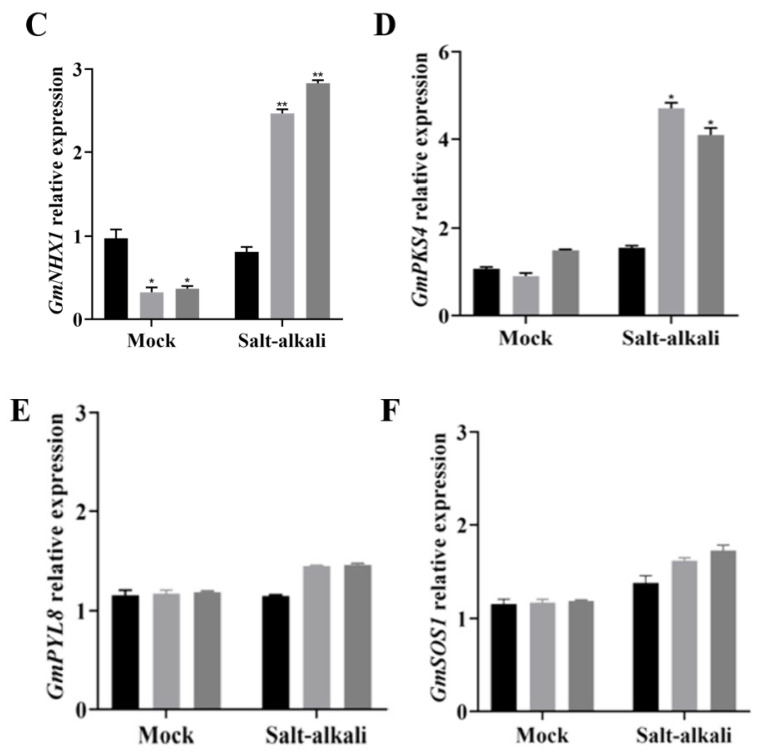
Using the expression level of the WT gene as the control group (with *GmActin* as the internal reference gene), the relative transcription levels of some stress-related genes in GmSNF4-transgenic soybeans. Expression of (**A**) *GmAKT1*, (**B**) *GmNCED3*, (**C**) *GmNHX1*, (**D**) *GmPKS4*, (**E**) *GmPYL8*, and (**F**) *GmSOS1* genes. The data represent the means and standard deviations of three repeats (*n* = 3). Significant differences were determined via unpaired two-sided Student’s *t*-tests (*, *p* < 0.05; **, *p* < 0.01).

The ABA-related genes *GmNCED3* and *GmPYL8*, ion-related genes *GmSOS1*, *GmAKT1*, and *GmNHX1*, and interaction gene *GmPKS4* in *GmSNF4* transgenic soybean plants and wild-type plants under salt–alkali stress were quantitatively analyzed using qRT-PCR (Figure 5A–F). This analysis was performed to determine the effect of the regulatory mechanism of the *GmSNF4* gene on the salt–alkali stress tolerance of soybean plants and to better understand the biological function of *GmSNF4*.

The results showed that the transcriptional levels of stress-related genes in OE plants were significantly higher than those in WT plants, indicating that the enhanced salt–alkali stress tolerance of OE plants may be related to the upregulated expression of these stress-related genes, with *GmNHX1* showing the most significant increase (Figure 5C). These findings suggest that the *GmSNF4* gene upregulates the transcription of stress-signal-related genes under salt–alkali stress.

### 2.5. Analysis of Stomatal Conductance of GmSNF4 Transgenic Soybean Leaves Under Salt–Alkali Stress

**Figure 6:**
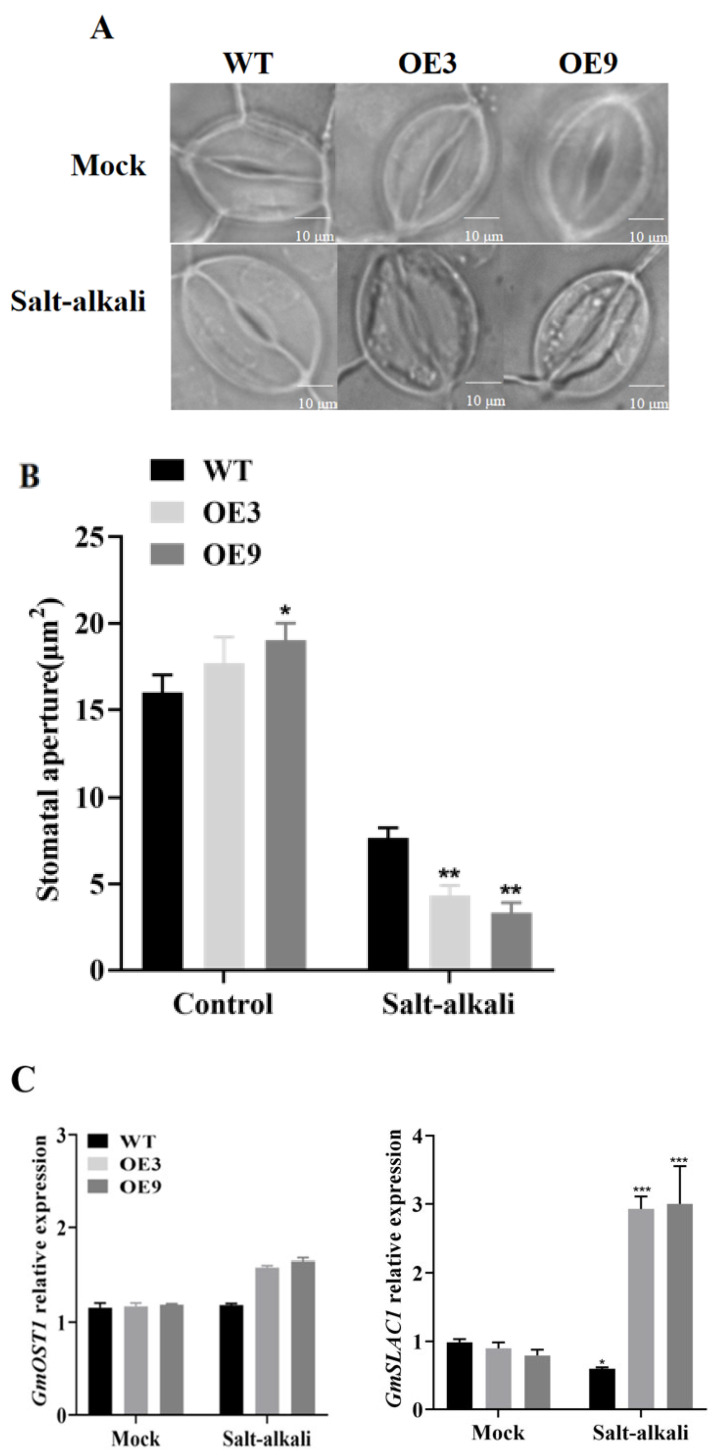
Effects of *GmSNF4* overexpression in transgenic soybean plants’ stomata pore size and gene expression. (**A**) Analysis of stomatal aperture of leaves before and after salt–alkali stress for soybean plants. The second trifoliate plants from the top were chosen for stoma observation. Bar = 10 μm. (**B**) The data represent the means ± standard deviations of 30 fields of view. Asterisks indicate significant differences between WT and transgenic plants under salt–alkali treatment conditions. Significant differences were determined via unpaired two-sided Student’s *t*-tests (*, *p* < 0.05; **, *p* < 0.01; ***, *p* < 0.001). (**C**) Analysis of the expression levels of *GmOST1* and *GmSLAC1* in soybean leaves transformed with the *GmSNF4* gene, using the WT gene expression level as the control group (with *GmActin* as the internal reference gene). The data are the means of ten biological replicates ± SDs (10 plants per replicate).

Stomata are not only the main medium for the exchange of carbon dioxide and water between plants and the air, but also the primary channel for maintaining the balance of the internal environments in plants [25]. To explore the effect of *GmSNF4* on stomatal movement, the stomatal aperture of transgenic plants under salt–alkali stress treatment was analyzed. As shown in Figure 6A,B, under normal growth conditions, the stomatal opening of OE and WT strains was similar. However, after 10 days of salt–alkali stress, the stomatal opening of OE plants was significantly lower than that of WT plants. To more intuitively observe these physiological changes, we conducted statistical analysis of the stomatal aperture area and the number of closed stomata (Figure 6B and Table S3). These results were consistent with the previously observed relative leaf water content and other physiological indices. Additionally, the expression levels of the stomata-related genes *GmOST1* and *GmSLAC1* were higher in transgenic plants than in wild-type plants under salt–alkali stress treatment (Figure 6C). Therefore, the *GmSNF4* gene can enhance the expression levels of stomata-related genes, thereby promoting stomatal closure and reducing water loss. This mechanism contributes to the plants’ resistance to stress damage [26].

### 2.6. Analysis of ROS (Reactive Oxygen Species) Content in Roots and Leaves of GmSNF4 Transgenic Soybean Under Salt–Alkali Stress

**Figure 7:**
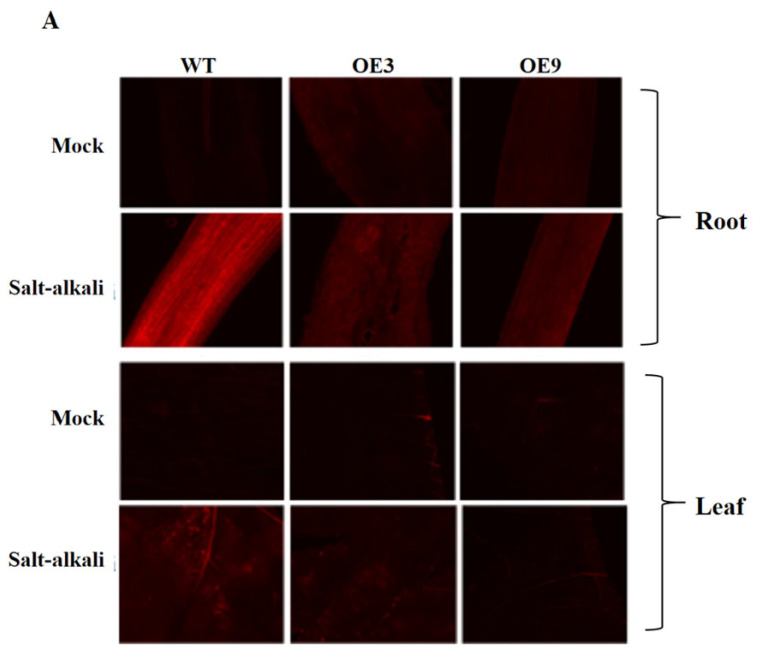

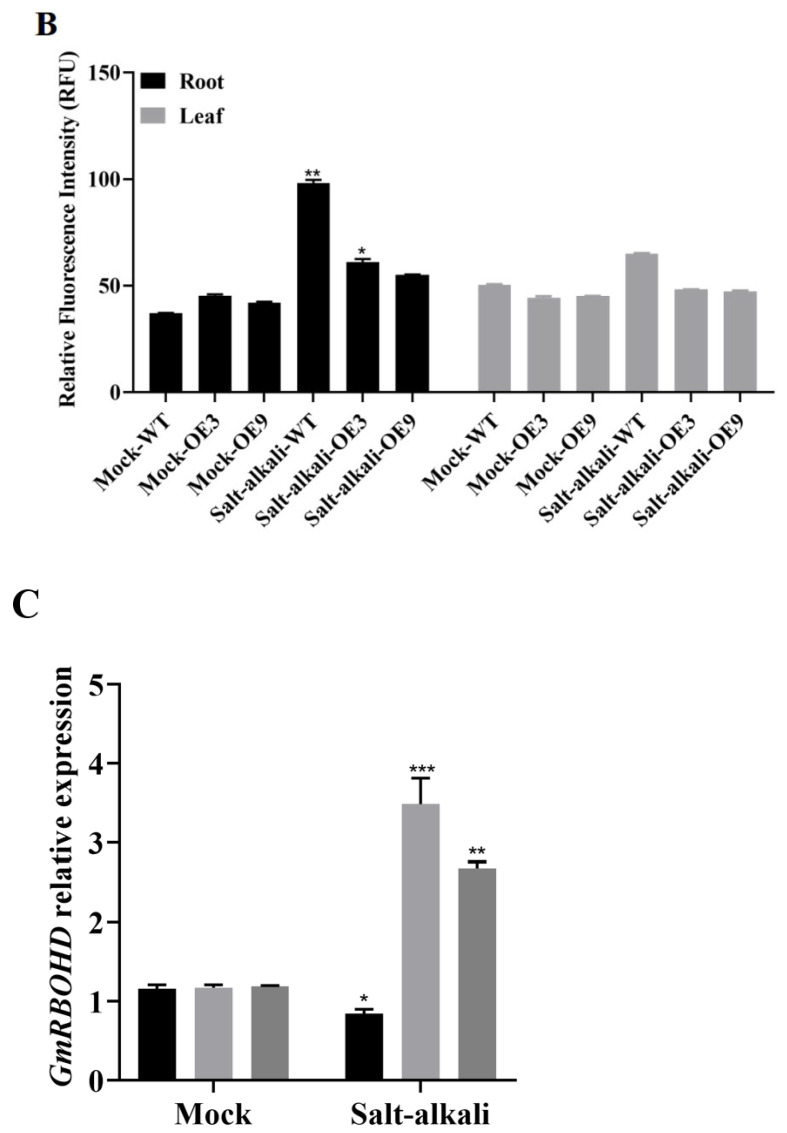
*GmSNF4* promotes ROS scavenging under salt–alkali stress. (**A**) H2DCF-DA (DCFH-DA) staining of NaCl+NaHCO_3_-induced ROS production in roots of *GmSNF4*. (**B**) Relative fluorescence intensity of soybean roots and leaves under salt–alkali stress. (**C**) Analysis of *GmRBOHD* expression levels in soybean leaves transformed with the *GmSNF4* gene, using WT gene expression levels as the control group (with *GmActin* as the internal reference gene). The data are the means of ten biological replicates ± SDs (10 plants per replicate). Significant differences were determined via unpaired two-sided Student’s *t*-tests (*, *p* < 0.05; **, *p* < 0.01; ***, *p* < 0.001).

A large number of studies have shown that a low concentration of reactive oxygen species (ROS) can act as a second messenger to mediate multiple responses in plant cell signal transduction pathways. However, high concentrations of ROS not only cause oxidative damage to plants but also lead to the formation of cytotoxic malondialdehyde, resulting in the inhibition of normal metabolism in plants [27,28,29]. To study the effect of the *GmSNF4* gene on ROS levels, the content of reactive oxygen species (ROS) in the roots and leaves of transgenic soybean plants under salt–alkali stress was detected, and the expression of the related gene *GmRBOHD* was quantitatively analyzed. RBOHD is a key enzyme that produces ROS in plant cells, and its upregulation can significantly enhance ROS production [30]. Under environmental stress, the upregulation of RBOHD expression facilitates the plant’s adaptation to adverse conditions [30,31]. Under normal conditions, the ROS levels in the roots and leaves of OE plants were similar to those of wild-type plants. However, under salt–alkali stress, the fluorescence signals of ROS in transgenic soybean plants were significantly weaker than those in wild-type plants (Figure 7A,B). This finding was consistent with the observed effects of hydrogen peroxide, superoxide anions, and malondialdehyde under salt–alkali stress (Figure 4A,B,I). Although the relative expression of *GmRBOHD* in OE soybean leaves increased under salt–alkali stress (Figure 7C), the fluorescence results indicated lower ROS levels. These results suggest that the *GmSNF4* gene reduces ROS content, thereby mitigating oxidative damage and enhancing the salt–alkali stress resistance of plants.

## 3. Discussion

Molecular genetic research on mutants has shown that the CBL-CIPK mechanism plays a key role in plant responses to various abiotic stresses, including drought, salt, and temperature stress [32,33,34,35,36]. *GmPKS4*, as a member of the CIPK family, participates in the CBL-CIPK mechanism to regulate ion balance and signal transduction [37]. *GmSNF4*, belonging to the SnRK1 subfamily, interacts with GmPKS4 to jointly regulate energy metabolism and ion transport. This interaction may integrates the CIPK and SnRK1 signaling pathways, thereby enhancing plant stress tolerance [38]. For example, studies have found that *GmSnRK1.1* and *GmSnRK1.2* show significant responses to alkaline stress in soybeans, and overexpression of these genes can enhance the alkaline tolerance of soybeans [39]. *GmPKS4* may function as an upstream kinase that phosphorylates the threonine residue in the T-loop of *GmSnRK1’s* catalytic subunit to activate it. Activated *GmSnRK1* may then phosphorylate specific Ser/Thr residues of *GmPKS4*, modulating its kinase activity or subcellular localization through a feedback loop. Additionally, *GmSNF4* (the βγ subunit of SnRK1) could sense cellular energy status via binding to AMP/ATP or sugar phosphates (such as trehalose-6-phosphate T6P), triggering activating conformational changes in the SnRK1 complex under salt–alkali stress. This enhances *GmPKS4*-mediated phosphorylation of *GmSnRK1*, thereby enabling the complex to synergistically regulate adaptive responses. Such synergistic interactions provide a new perspective for understanding the mechanisms of plant salt–alkali tolerance.

High salinity and high alkalinity can cause ionic stress in plants [40]. An excessive Na^+^ concentration disrupts ion homeostasis both inside and outside plant cells, leading to the excessive accumulation of Na^+^ and a reduction in K^+^ content [41]. The dynamic balance of intracellular K^+^/Na^+^ is primarily maintained through the regulation of ion channels and transporters. According to existing studies, the CBL-CIPK mechanism can regulate ion transport under salt–alkali conditions. The CBL-CIPK module plays an important role in calcium signaling pathways, where calcium acts as a second messenger, especially in regulating the activity of ion transporters in response to various abiotic stresses. For example, hypokalemia stress may trigger cytoplasmic calcium signaling, which activates CIPK23 via CBL1 and CBL9, and subsequently phosphorylates and activates the potassium channel AKT1 [42,43,44]. In the current study, SOS1 and NHX1 were identified as key determinants of cellular Na^+^ homeostasis: SOS1 controls the net efflux of Na^+^ through the plasma membrane, while NHX1 controls the sequestration of free Na^+^ into the vacuole through the vacuolar membrane [45]. The plasma membrane localization of the Na^+^/K^+^ exchanger SOS1 in *Arabidopsis* facilitates Na^+^ efflux under high-salt conditions [46]. NHX1 affects H^+^ transport by sequestering Na^+^ into the vacuole and transporting H^+^ into the cytoplasm across the vacuolar membrane [47]. Therefore, the SOS1 and NHX1 genes are crucial for plant responses to adverse stress conditions. In this study, the expression of *GmSOS1* and *GmNHX1* in transgenic leaves increased under salt–alkali stress (Figure 5C,F), demonstrating that the *GmSNF4* gene affects intracellular ion homeostasis and enhances soybean salt–alkali stress tolerance.

After plants are stimulated by other adverse conditions such as low or high temperature, drought, or salt stress, a large amount of abscisic acid is synthesized within the plant, leading to the closure of stomata, which plays a protective role [48]. In the current study, the slow anion channel SLAC1 was found to play a key role in the stomatal closure process by sensing external signals and altering its molecular conformation, thereby closing the stomata [49]. OST1 can phosphorylate the anion channel SLAC1 and regulate ABA-mediated stomatal closure [50]. This study confirmed that under salt–alkali stress, the expression of both *GmOST1* and *GmSLAC1* increased (Figure 6C). Specifically, the expression of *GmSLAC1* increased significantly, and the state of stomatal closure was consistent with the known research findings (Figure 6A,C).

The expression of ABA-related genes *GmNCED3* and *GmPYL8* also increased (Figure 5B,E). Overexpression of the ABA dioxygenase NCED3 in plants also increases endogenous ABA levels [51]. This increase in ABA levels promotes stomatal closure, thereby enhancing plant tolerance to stress environments. In the presence of ABA, the ABA receptor PYL8 can interact with PP2C-class phosphatases. The bound protein kinase SnRK2 (sucrose non-fermenting-1-related protein kinase 2) becomes dissociated from PYL8, allowing it to autophosphorylate, regain activity, and then further phosphorylate downstream genes such as ABF2 [52]. SnRK2 and ABFs (ABA response element [ABRE] binding factors) are core components of the ABA signaling pathway involved in the drought stress response. Experiments have shown that PtrSnRK2.4, a member of the PtrSnRK2 family, is strongly induced by ABA and can interact with and phosphorylate *PtrABF2* [53]. Experimental analysis indicates that the ability of *PtrABF2* to bind to the transcriptional promoter of its target gene *PtrADC* in Poncirus trifoliata depends on the phosphorylation modification of PtrSnRK2.4. This phosphorylation enables *PtrABF2* to positively regulate *PtrADC* under drought stress. Therefore, the upstream protein kinase PtrSnRK2.4 phosphorylates *PtrABF2*, mediating the synthesis and accumulation of putrescine in plants [53]. The above results demonstrate that the *GmSNF4* gene reduces damage to plants by increasing the expression of key genes related to stomatal closure and ABA signaling.

Under non-stress conditions, reactive oxygen species (ROS) levels are low, but abiotic stress increases ROS, causing cellular damage. Plants activate antioxidant systems involving SOD, CAT, and POD to maintain ROS homeostasis [54]. In this study, overexpression of GmSNF4 enhanced the antioxidant system, reducing MDA levels and increasing proline content under salt–alkali stress (Figure 4G–J). DAB and NBT staining showed less damage in overexpressing soybean leaves, and fluorescence intensity indicated reduced ROS accumulation (Figure 4C,D and Figure 7A). These results suggest that *GmSNF4* enhances salt–alkali stress tolerance by strengthening the antioxidant system and protecting plants from oxidative and osmotic stress.

## 4. Materials and Methods

### 4.1. Yeast Two-Hybrid (Y2H) Test

The interaction between GmSNF4 and GmPKS4 in yeast cells was detected using the yeast two-hybrid (Y2H) method. The coding sequences of GmSNF4 and GmPKS4 were inserted into the pGADT7 and pGBKT7 vectors, respectively. The constructed vectors were then co-transformed into competent Y2HGold yeast cells and plated on SD/-Leu/-Trp double dropout medium. After colony growth, a single colony was selected and cultured until it reached an OD_600_ of 0.8. Next, 2 μL of the bacterial solution was spotted on both SD/-Leu/-Trp double dropout medium and SD/-Trp/-Leu/-His/-Ade + X-α-Gal quadruple dropout medium. The colonies were then incubated at 29 °C for 5–7 days and observed.

### 4.2. Bimolecular Fluorescence Complementarity (BiFC)

The interaction between GmSNF4 and GmPKS4 was observed using the BiFC (Bimolecular Fluorescence Complementation) method. The target gene fragments of GmSNF4 and GmPKS4 were inserted into the pXY104 and pXY106 vectors, respectively, to construct the pXY104-GmSNF4 and pXY106-GmPKS4 fusion proteins. The constructed vectors were then transformed into *Agrobacterium tumefaciens* EHA105 and subsequently infiltrated into the abaxial (lower) side of tobacco leaves, which were then marked. After incubation at 21 °C for 48–72 h, the yellow fluorescence signal of YFP (Yellow Fluorescent Protein) was observed under a confocal laser scanning microscope.

### 4.3. Plant Genetic Transformation Methods

The genetic transformation methods of soybeans are as follows in three parts:

1. Place several plump “Dongnong 50” soybean seeds in a sterile Petri dish. Two other plates were coated with anhydrous calcium chloride. These plates and two bottles containing 96 mL of sodium hypochlorite were sealed in a container with petroleum jelly and plastic wrap. Concentrated hydrochloric acid (5 mL) was added to the bottles to generate chlorine gas for sterilization for 16 h. The seeds were then aired out with a fan for 30 min to remove excess chlorine gas and soaked in sterilized distilled water for one day.
2. A total of 100 µL of frozen bacterial suspension was spread on YEP solid medium with kanamycin and acetosyringone (AS) and incubated for 18 h at 28 °C. The bacterial suspension was scraped off and transferred to liquid LCCM infection solution, ensuring an OD_600_ value of 0.6–0.8. Sterilized soybeans were halved and wounded on the embryonic axis, then placed in the infection solution and shaken at 28 °C for 30 min. Excess solution was removed with filter paper, and the beans were placed on co-cultivation medium with the wounded side down and cultivated in darkness for three days.
3. Cotyledons of the seedlings were cut into thirds and inserted into SI-1 medium at a 45° angle for 7 days. They were then transferred to SI-2 medium for 14 days, repeating the process with fresh SI-2 medium. Afterward, the cotyledons were removed, and clustered buds were transferred to stem elongation medium for three weeks. When stems reached 5–6 cm, plants were transferred to rooting medium. After vigorous root and branch growth, plants were hardened off for 2 days before being transferred to soil and wrapped with plastic wrap for a week. Plants were tested once new leaves appeared and normal growth was confirmed [43].

Because *Arabidopsis thaliana* is highly sensitive to *Agrobacterium*, the floral dip method is often used to directly soak flowering plants in an *Agrobacterium* suspension. This simple and efficient process allows the acquisition of transgenic seeds without the need for tissue culture [43]. For soybeans, seedling leaves were selected for trace DNA extraction, and PCR identification was performed using primers designed for the *GmSNF4* gene. This approach enabled the identification of stable transgenic soybeans and *Arabidopsis*. After identifying 15 positive *Arabidopsis* lines (Figure S7) and 9 soybean lines transformed with the *GmSNF4* gene, the plants were cultivated to the T3 generation for subsequent experiments [20,55].

### 4.4. Plant Cultivation Methods

*Arabidopsis* seeds were vernalized in sterile water at a low temperature (4 °C) in the dark for 2–3 days. Sterilized vermiculite and nutritious soil were mixed at a ratio of 8:2 and used to cultivate the seedlings under low light conditions for 3–4 days. When the *Arabidopsis thaliana* seedlings had grown three pairs of leaves, they were transplanted and grown for an additional 3–4 weeks before being used in the experiment.

After vernalization for two days, *Nicotiana benthamiana* seeds were sown on a 1:1 mixture of sterilized nutritious soil and vermiculite and cultured under low light conditions for 3–4 days. The tobacco seedlings were then allowed to grow for an additional three weeks until they had developed two leaves, at which point they were transplanted. The growth conditions were as follows: sufficient watering, a photoperiod of 14 h light/10 h darkness, a temperature of 25 °C, and a relative humidity of 70% in the artificial climate chamber.

The *GmSNF4* transgenic soybean seeds and corresponding wild-type (WT) soybean seeds were planted in pots containing vermiculite, with four seeds per pot. The pots were placed in an artificial climate chamber maintained at 23 °C and 50% relative humidity, with a photoperiod of 16 h of light and 8 h of darkness. Initially, 2–3 soybean seeds were germinated in the dark and then cultivated in a low-light environment. The light intensity was gradually increased to allow the soybean seedlings to grow normally. During this period, all pots were watered sufficiently. When the seedling develops its fourth true leaf, subject it to stress treatment.

### 4.5. Plant Exposure to Salt-Alkali Stress

After the fourth true leaf of the soybean seedlings emerged, the overexpressing (OE) soybeans and the vector controls were subjected to the following salt–alkali stress treatment: On the first, second, and fifth days, 100 mL of the following solutions were applied, respectively: 60 mM NaCl + 40 mM NaHCO_3_, 70 mM NaCl + 50 mM NaHCO_3_, and 90 mM NaCl + 60 mM NaHCO_3_. For the control group, 100 mL of water was added at each of the three time points. The phenotypic changes of the soybean plants were observed daily and photographed.

The *GmSNF4-OE Arabidopsis* and vector control seedlings were subjected to salt–alkali stress treatment as follows: On the first, second, and fifth days, 50 mL of the following solutions were applied, respectively: 30 mM NaCl + 20 mM NaHCO_3_, 60 mM NaCl + 30 mM NaHCO_3_, and 90 mM NaCl + 40 mM NaHCO_3_. For the control group, 50 mL of water was added at each of the three time points. The phenotypic changes in the *Arabidopsis* seedlings were observed daily and photographed.

### 4.6. RNA Extraction, cDNA Synthesis, and Real-Time Quantitative PCR (RT-qPCR)

RNA was extracted from the first pair of leaves of both the vector control and *GmSNF4-OE* soybeans. RNAiso Plus reagent (TaKaRa) was used for RNA extraction. All materials used in the extraction process, including centrifuge tubes, micro-pipette tips, and mortars, were required to be RNA-free to avoid contamination. The extracted RNA was reverse-transcribed into cDNA using the Transcript All-in First-Strand cDNA Synthesis SuperMix kit (TransGen Biotech, Beijing, China). Gene-specific primers were designed using Primer 5.0 software. Real-time PCR analysis was performed using Taq SYBR Green qPCR Premix (Servicebio, Wuhan, China). The actin gene was used as an internal reference to normalize the expression levels. In this study, the relative expression level of the target gene was analyzed by the 2^−ΔΔCT^ method [1].

### 4.7. Analysis of Subcellular Localization

The green fluorescent protein (GFP) fusion expression vector pCAMBIA1302-GmSNF4 was constructed using the electrotransformation method. The vector was then transferred into *Agrobacterium tumefaciens* EHA105 and incubated at 29 °C for two days. The transformed bacteria were subsequently transferred into 10 mL of YEB liquid culture medium. The bacterial culture was centrifuged at 4000 rpm for 4 min, and the pellet was resuspended in 10 mM MgCl_2_ solution containing 120 µM acetosyringone (AS). The optical density at 600 nm (OD_600_) was adjusted to approximately 0.6. Healthy tobacco plants were selected, and the bacterial suspension was injected into the abaxial (lower) epidermis of the leaves using a 1 mL syringe with a needle. The injected sites were marked, and the plants were cultured under low light conditions for 2 days. The fluorescence signal was then detected using a laser confocal microscope. The excitation wavelength used was 488 nm, and the emission wavelength was observed at 507 nm for the tobacco mesophyll cells.

### 4.8. Functional Analysis of Promoter

Using soybean genomic DNA as a template, a nucleic acid sequence of 1495 bp upstream of the *GmSNF4* gene ATG was obtained through PCR amplification (Figure S7). The *GmSNF4* gene promoter was then replaced via double-enzyme digestion with restriction enzymes Hind III and Xba I and subsequently inserted upstream of the GUS gene in the pCAMBIA3301 vector. The recombinant plasmid, designated as pCAMBIA3301-GmSNF4::GUS, was successfully constructed. The recombinant plasmid was transferred into *Agrobacterium tumefaciens* EHA105 using the standard Agrobacterium-mediated transformation method. *Arabidopsis thaliana* was then genetically transformed using the floral dip method. The constructed vector was introduced into wild-type *Arabidopsis* plants via *Agrobacterium* EHA105, and transgenic plants were selected through screening. After the T1 generation *Arabidopsis* plants were identified by PCR, T2 generation seedlings containing the *GmSNF4* gene were isolated and tested. GUS histochemical detection was performed to verify the expression of the GUS reporter gene. For the analysis of the regulatory ability of the *GmSNF4* promoter, both transgenic *Arabidopsis thaliana* and wild-type *Arabidopsis thaliana* with high expression and stable inheritance of the *GmSNF4* gene were subjected to salt–alkali stress treatment (120 mM NaCl + 80 mM NaHCO_3_) and then treated with GUS buffer [0.1 M Na_2_HPO_4_, 0.5 mM K_4_Fe(CN)_6_, 0.5 mM K_3_Fe(CN)_6_, 10 mM Na_2_EDTA, 0.06% Triton X-100]. The samples were rinsed 2–3 times, and GUS staining solution (GUS buffer and 0.5 mg/mL 5-bromo-4-chloro-3-indolyl glucuronide, pH 7.0) was added to ensure that the seedlings were completely soaked in the dye solution and stained for 24 h. The treated plants were washed and decolorized with anhydrous ethanol, followed by several rinses with distilled water under a microscope. The regulatory ability of the *GmSNF4* promoter was analyzed via GUS staining (Figure S8).

### 4.9. Determination of Phenotype and Physiological Indices

Soybean plants that had developed their first pair of leaves at the small clover stage were subjected to salt–alkali stress. After 10 days of treatment, leaves were collected to determine the following physiological indices: MDA (malondialdehyde), PRO (proline), SOD (superoxide dismutase), POD (peroxidase), CAT (catalase), H_2_O_2_ (hydrogen peroxide), and O_2_^•−^ (superoxide anion). All the above physiological indicators were tested using a Nanjing Jiancheng Kit. Additionally, reactive oxygen species (ROS) levels were measured, followed by stomatal analysis.

To intuitively and accurately reflect the degree of damage and accumulation of hydrogen peroxide (H_2_O_2_) and superoxide anions (O_2_^•−^) in leaves, DAB (3,3′-diaminobenzidine) and NBT (nitroblue tetrazolium) staining were performed. Leaves of the same size from the same parts of wild-type (WT) and transgenic seedlings were collected. The leaves were washed, dried, and placed in a 10 mL centrifuge tube for staining. After staining, the leaves were decolorized with anhydrous ethanol. Color changes were observed and photographed [27].

The content of ROS was determined using the red fluorescent probe dihydroethidium (DHE) method. Roots and leaves were placed in a ROS staining solution (PBS buffer at a 1:1000 dilution). Plant samples were incubated at room temperature for 60 min, and images were captured under a fluorescence microscope with an excitation wavelength of 543 nm.

Fresh leaves were decolorized in a mixture of acetic acid and ethanol (ethanol/acetic acid = 6:1 *v*/*v*) for 24 h. The leaves were then dehydrated with 70% ethanol, repeated 2–3 times for 0.5 h each. Finally, the leaves were treated with a clearing solution (8:1:2 chloral hydrate/glycerol/water) for 12 h. Images were observed and recorded under a microscope.

### 4.10. Statistical Analysis

All experiments were performed in triplicate. Graphs were prepared using GraphPad Prism 8. Specifically, unpaired two-tailed Student’s *t*-tests were used to determine the significance of the differences (*, *p* < 0.05; **, *p* < 0.01; ***, *p* < 0.001).

## 5. Conclusions

In this study, the interaction between GmSNF4 and GmPKS4 was identified using the yeast two-hybrid system and the BiFC (Bimolecular Fluorescence Complementation) technique. The *GmSNF4* gene was found to positively affect salt–alkali stress tolerance, and its encoded protein was localized in both the nucleus and cytoplasm. Subsequently, the function of the *GmSNF4* gene in soybeans was further analyzed. Overexpression of *GmSNF4* in plants increased the expression of key genes involved in stomatal regulation and reactive oxygen species (ROS) metabolism, as well as several stress-related genes. This led to stomatal closure, reduced ROS content, and enhanced salt–alkali stress tolerance. The results of this study indicate that *GmSNF4* can serve as a valuable genetic resource for plant breeding and lay the foundation for further research into its signaling pathway.

## Data Availability

The data are unavailable due to privacy.

## Supplementary Materials

The following supporting information can be downloaded at: https://www.mdpi.com/article/10.3390/plants14142218/s1.
