# Supplementary figures and images for: Soybean GmSNF4 Confers Salt–Alkali Stress Tolerance in Transgenic Plants
Source: Plants (Basel). 2025 Jul 17;14(14):2218. doi: 10.3390/plants14142218 (PMC12297902; doi:10.3390/plants14142218)

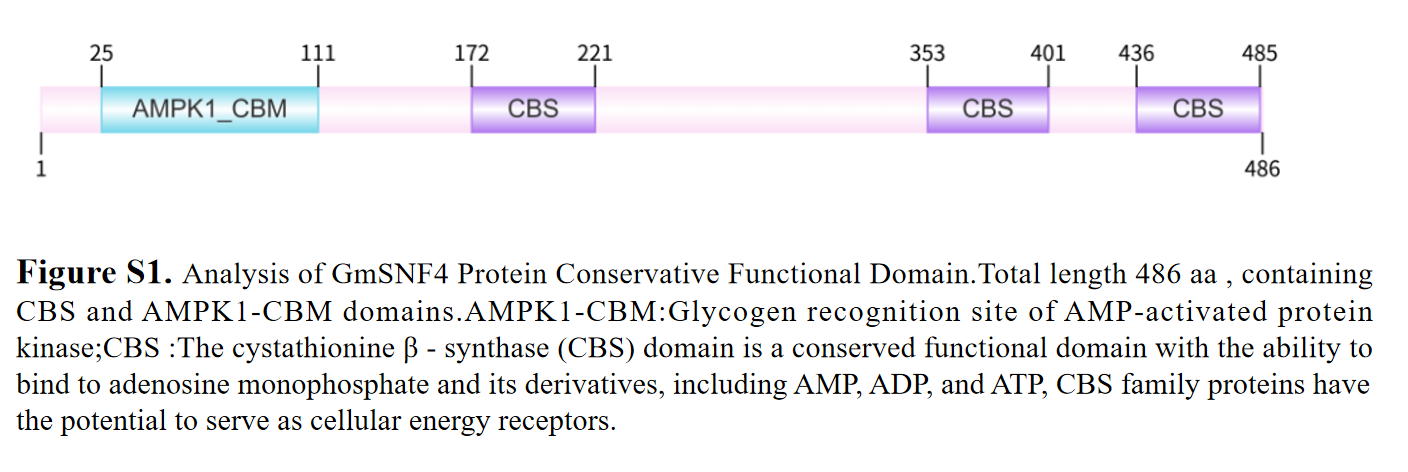

Supplement: Supplementary file 1 [file plants-14-02218-s001.zip › Figure S1.png]

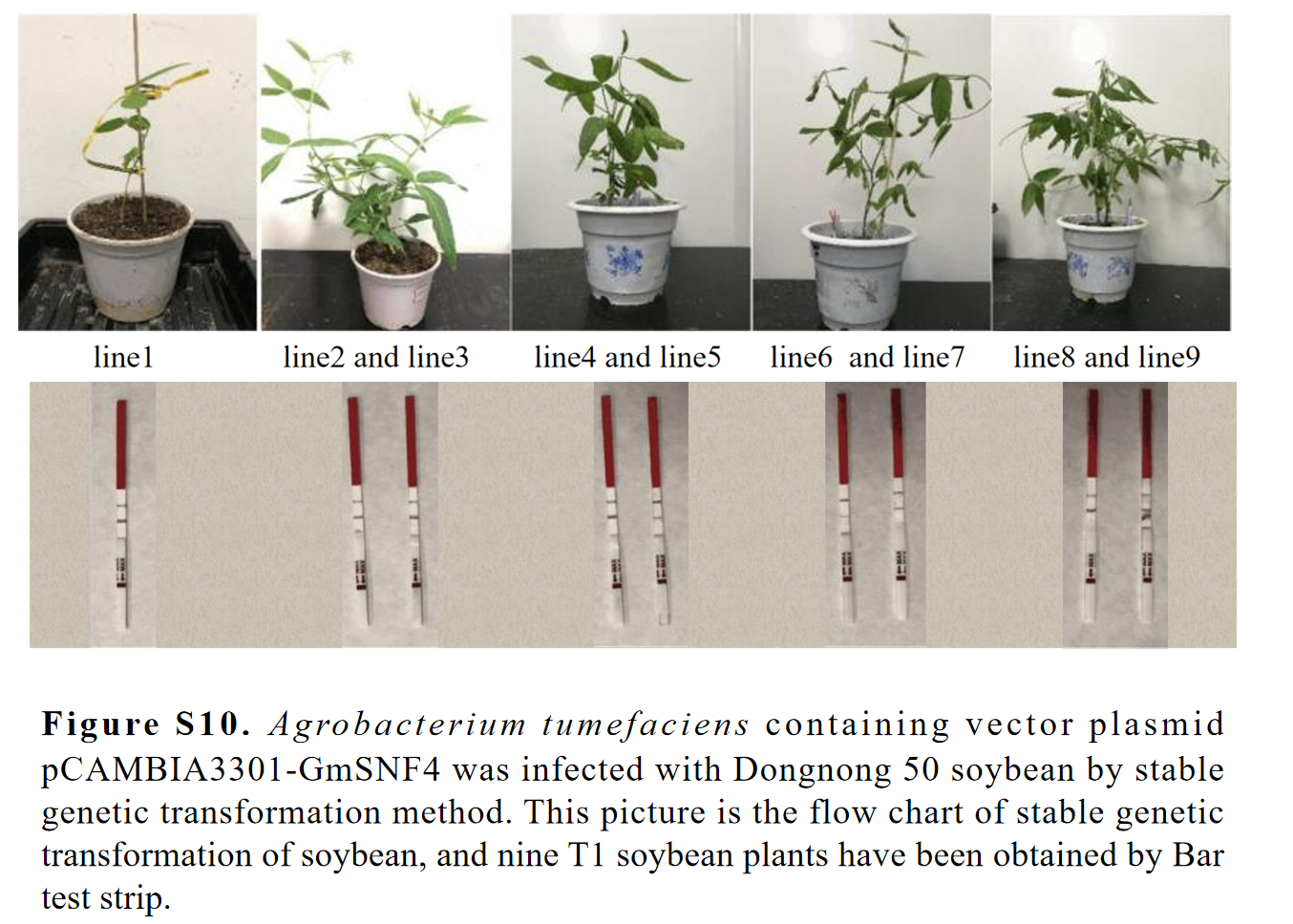

Supplement: Supplementary file 1 [file plants-14-02218-s001.zip › Figure S10.png]

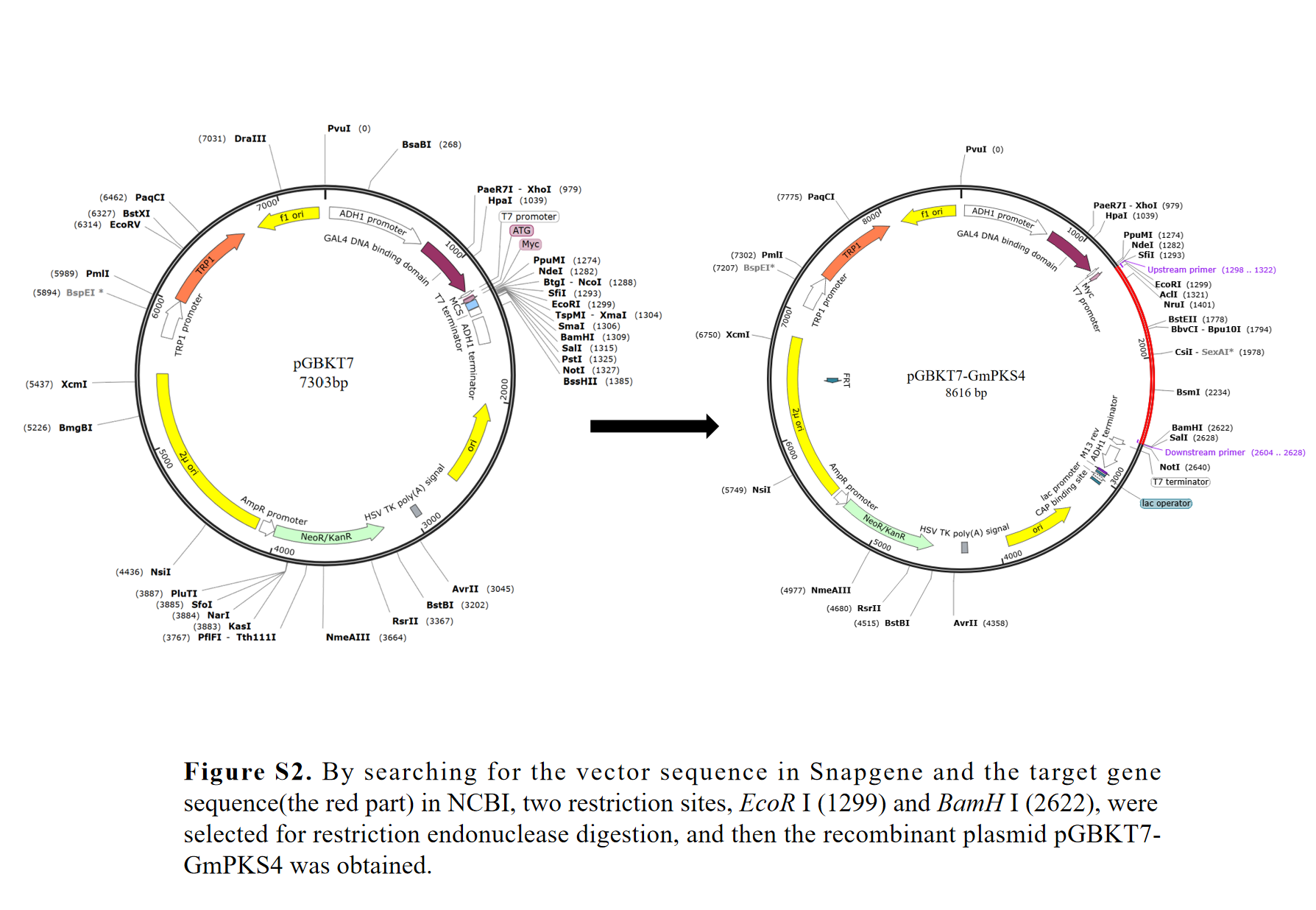

Supplement: Supplementary file 1 [file plants-14-02218-s001.zip › Figure S2.png]

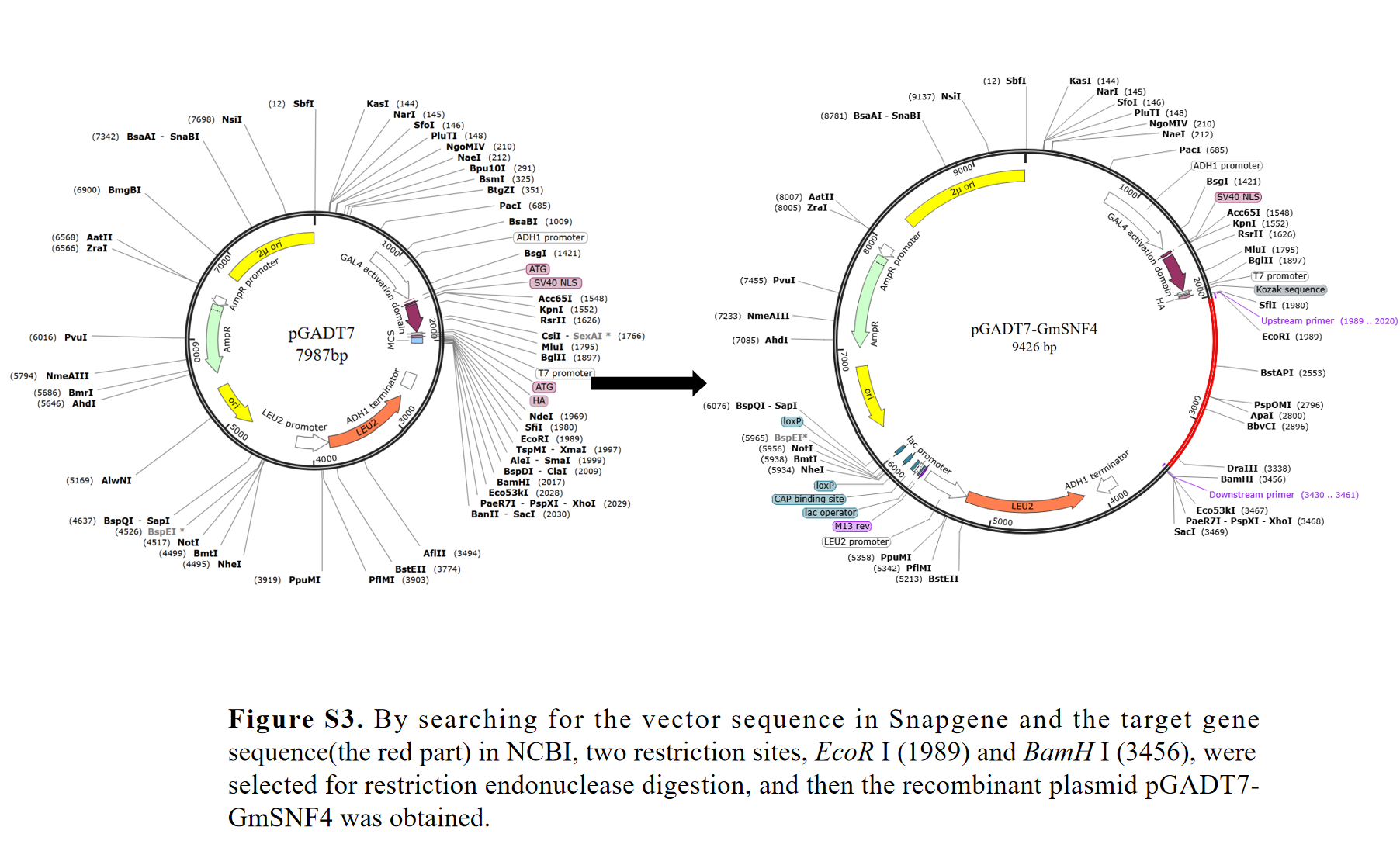

Supplement: Supplementary file 1 [file plants-14-02218-s001.zip › Figure S3.png]

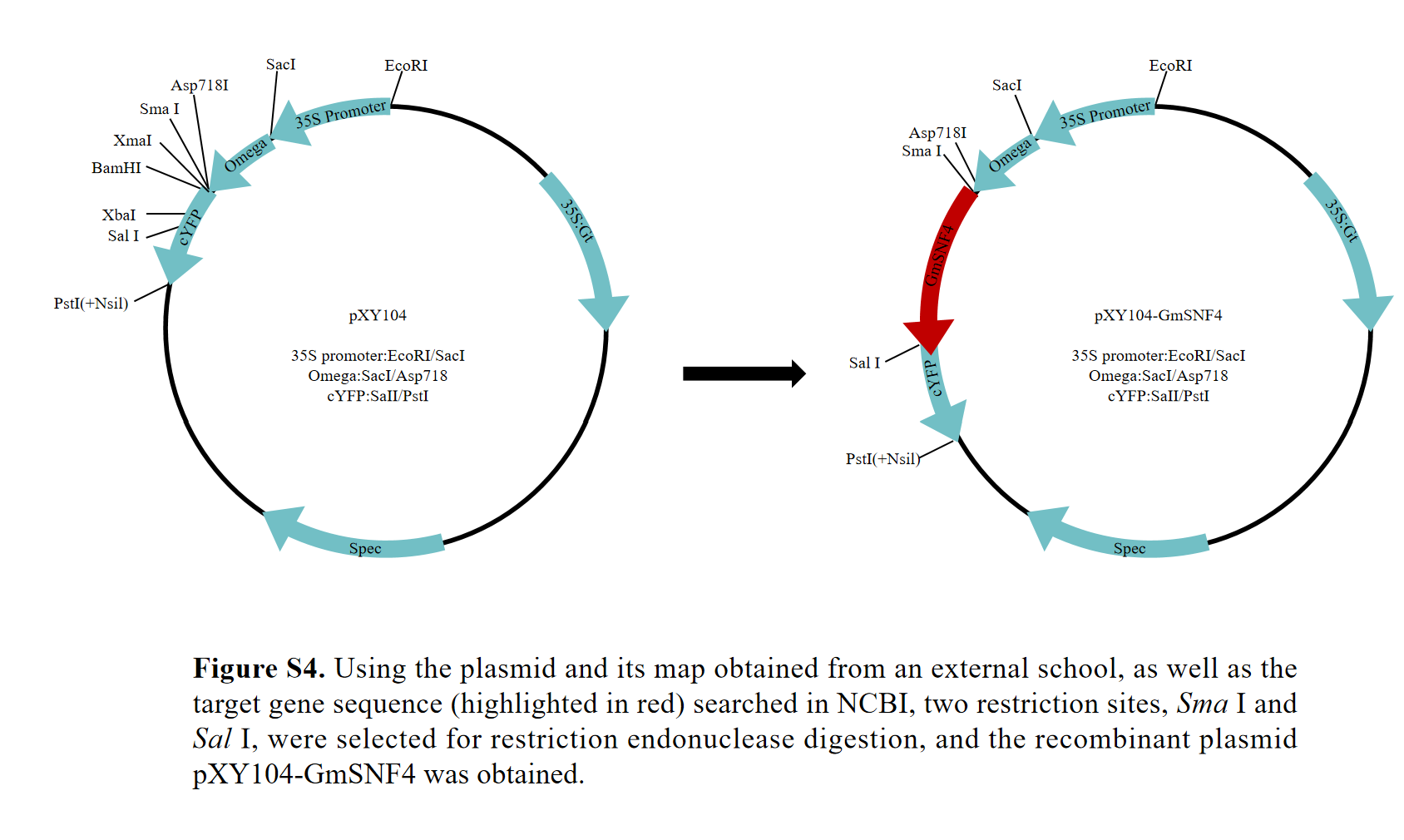

Supplement: Supplementary file 1 [file plants-14-02218-s001.zip › Figure S4.png]

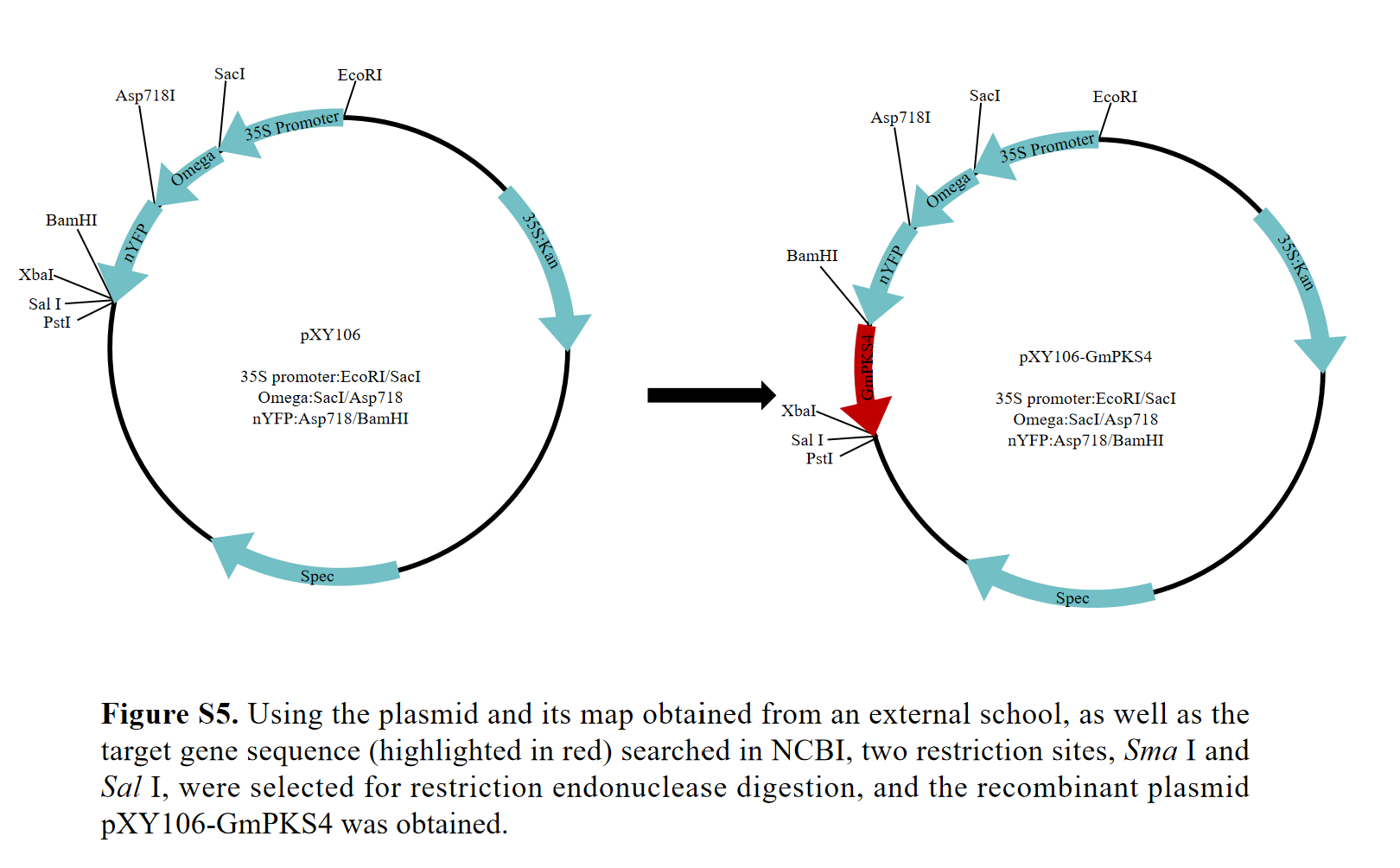

Supplement: Supplementary file 1 [file plants-14-02218-s001.zip › Figure S5.png]

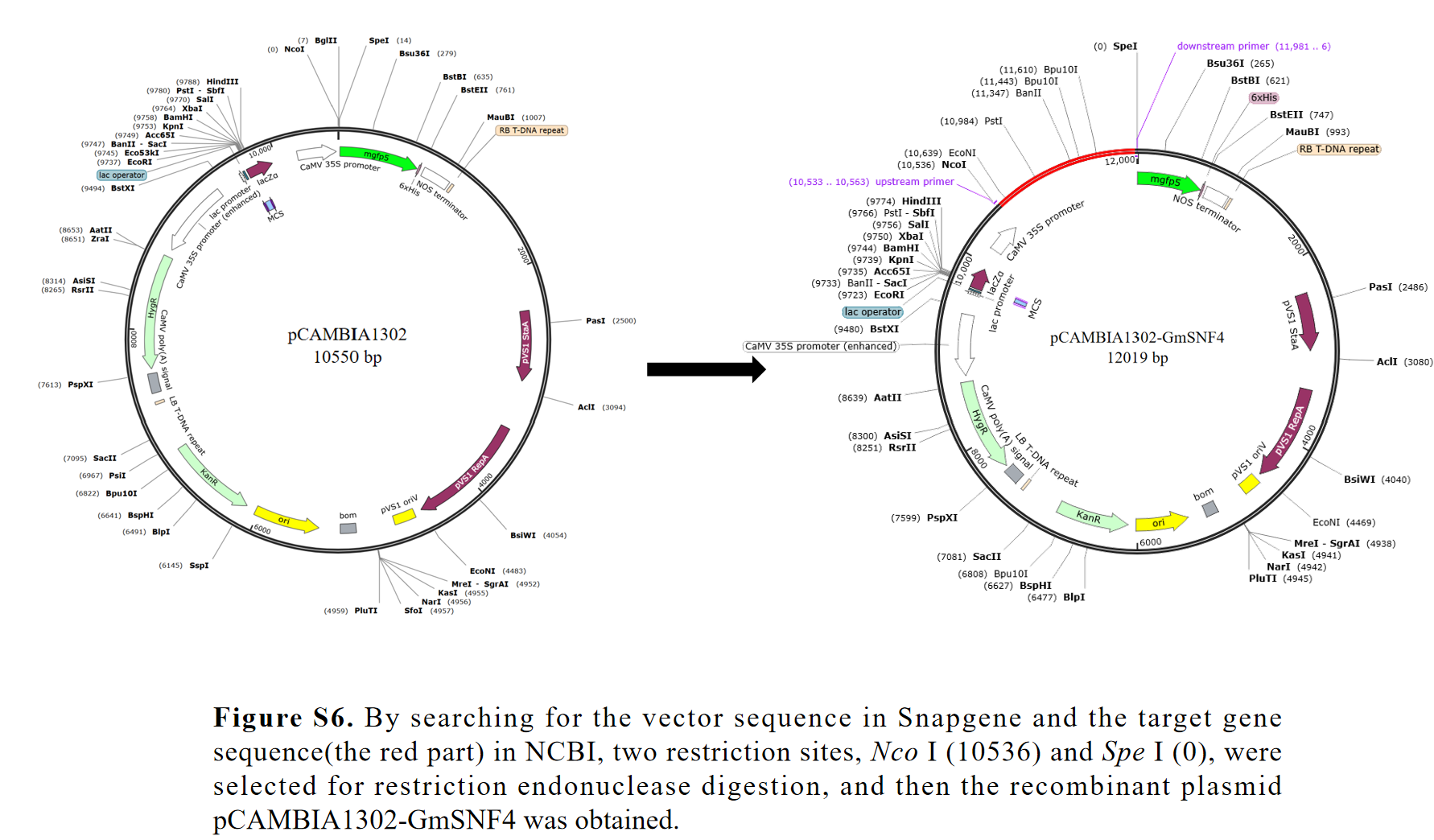

Supplement: Supplementary file 1 [file plants-14-02218-s001.zip › Figure S6.png]

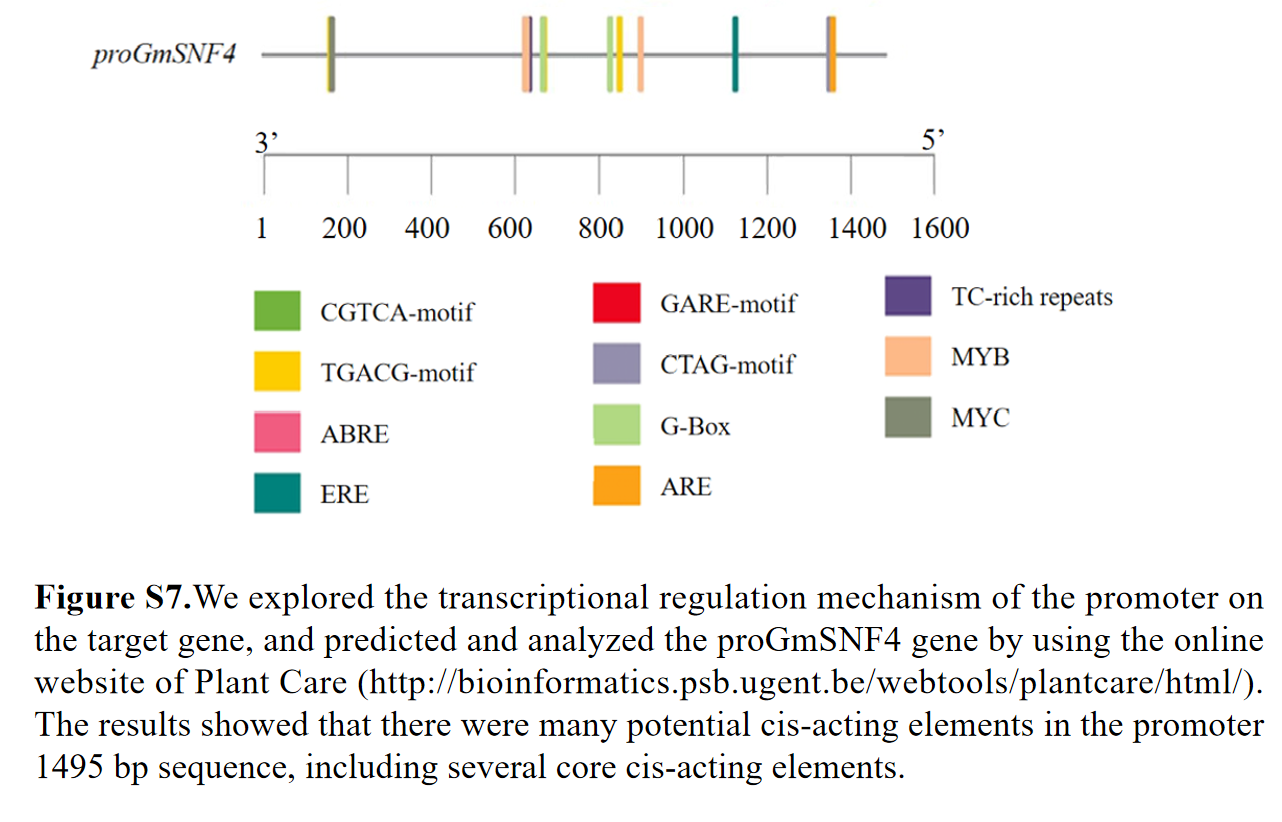

Supplement: Supplementary file 1 [file plants-14-02218-s001.zip › Figure S7.png]

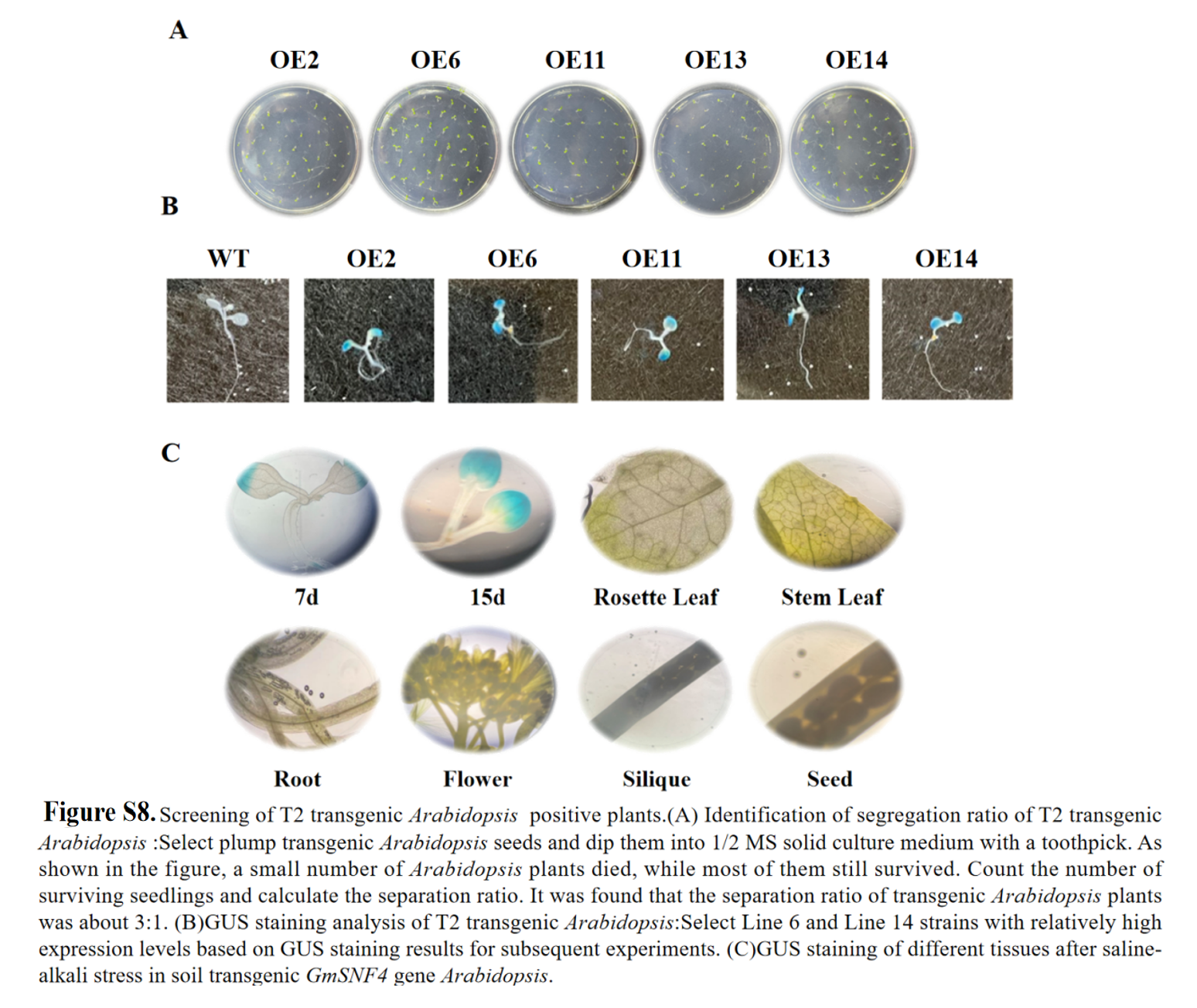

Supplement: Supplementary file 1 [file plants-14-02218-s001.zip › Figure S8.png]

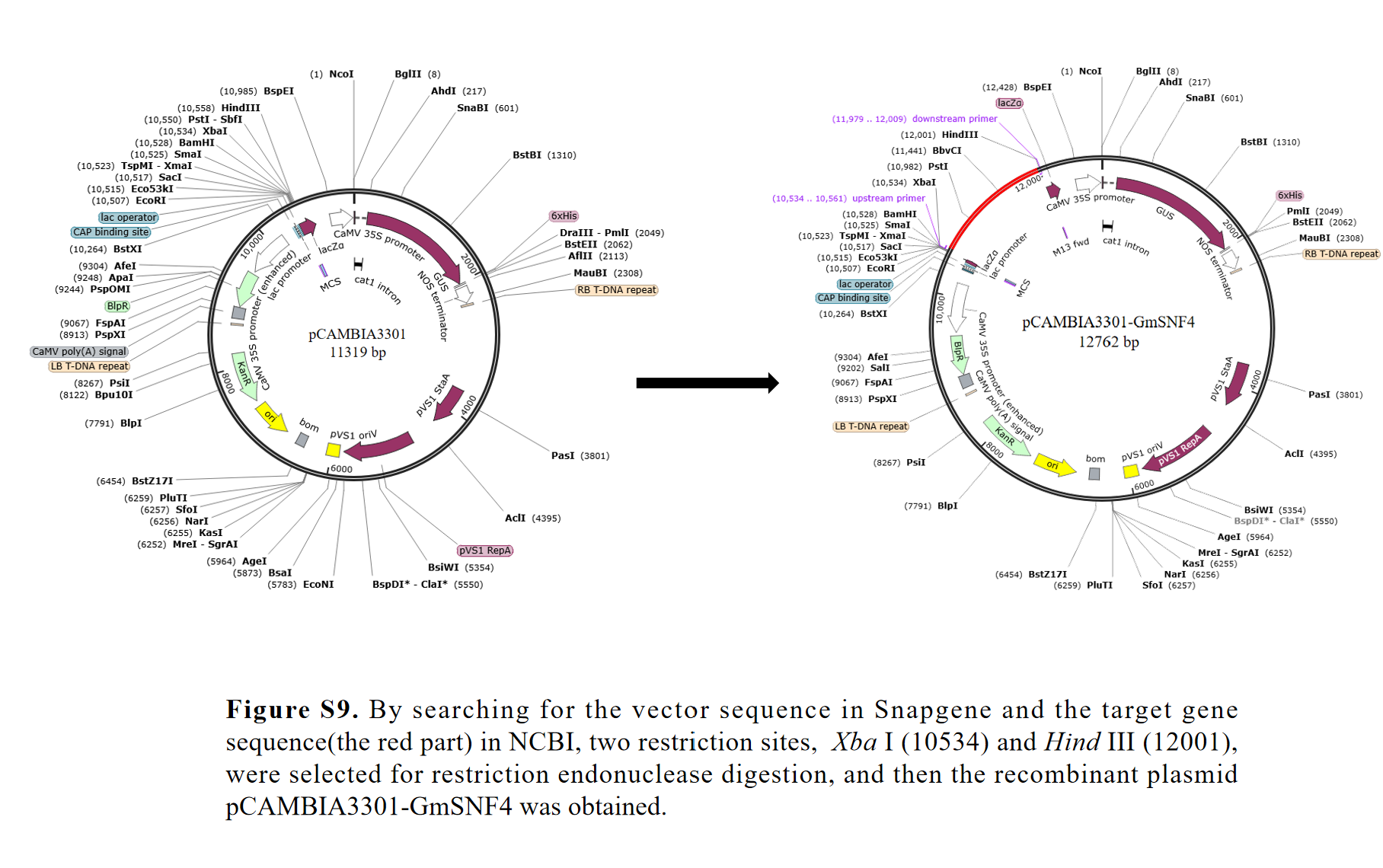

Supplement: Supplementary file 1 [file plants-14-02218-s001.zip › Figure S9.png]

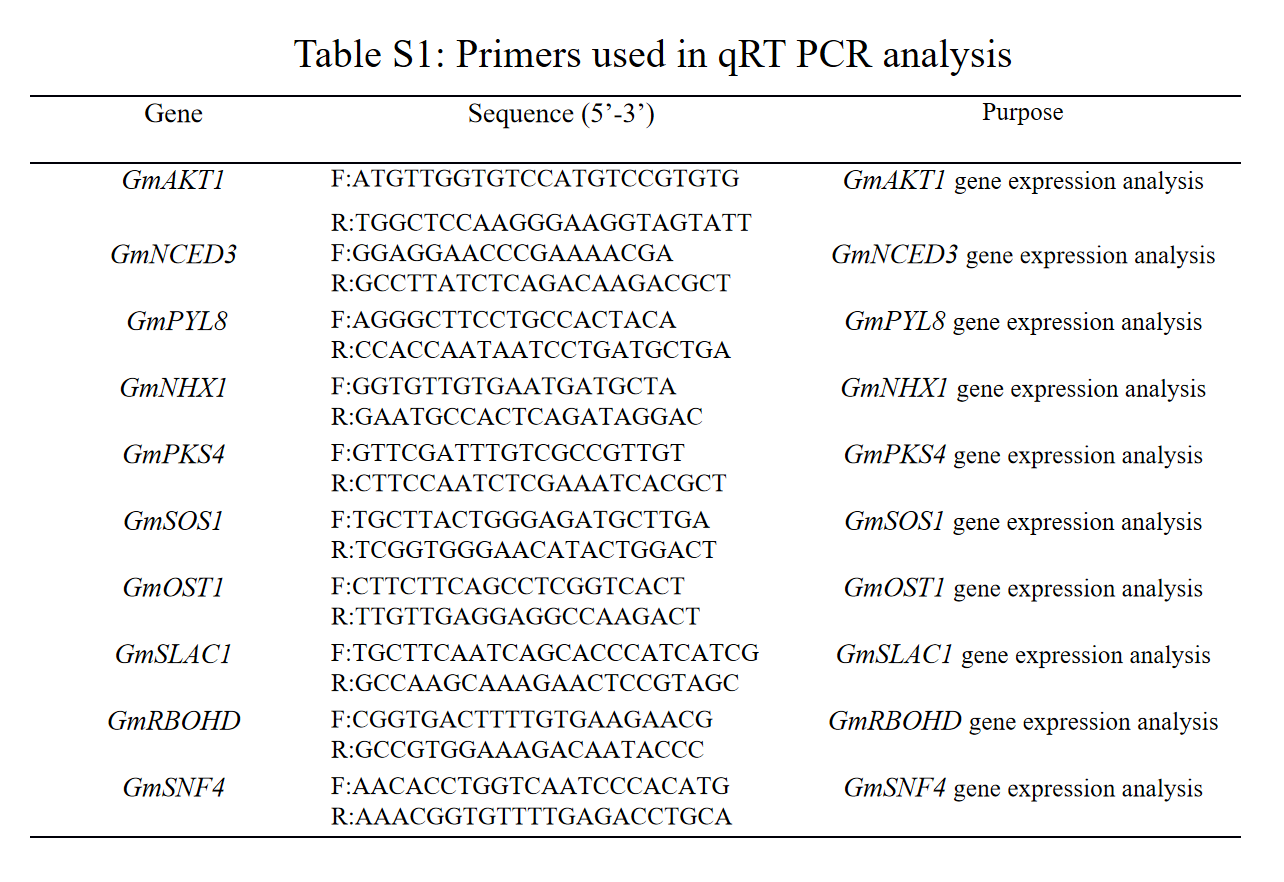

Supplement: Supplementary file 1 [file plants-14-02218-s001.zip › Table S1.png]

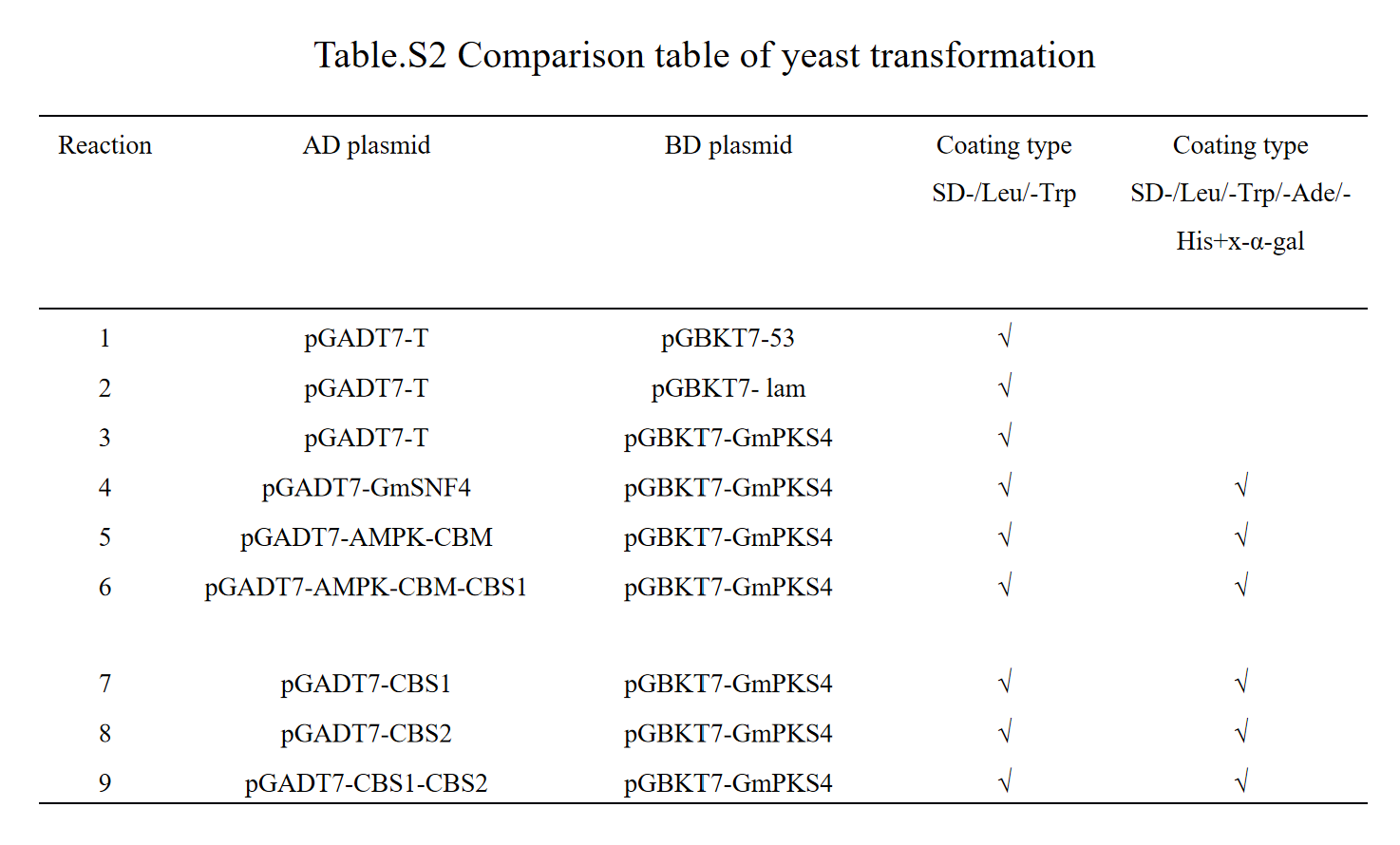

Supplement: Supplementary file 1 [file plants-14-02218-s001.zip › Table S2.png]

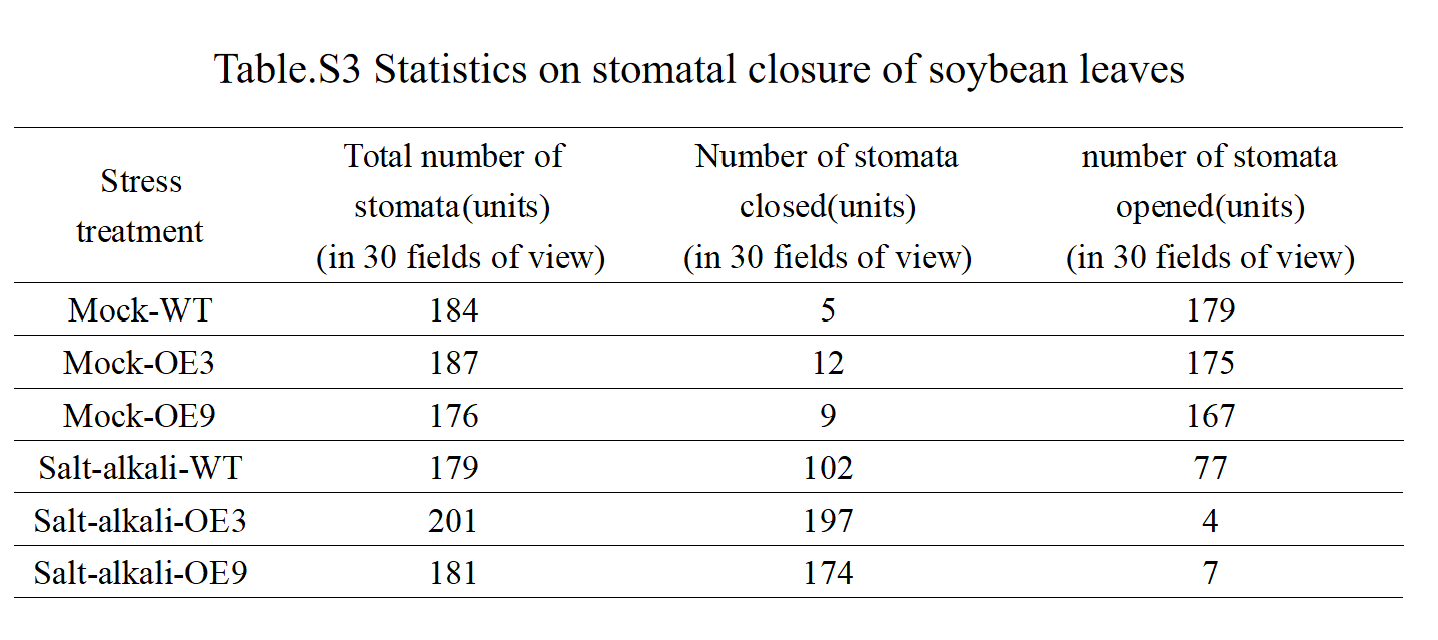

Supplement: Supplementary file 1 [file plants-14-02218-s001.zip › Table S3.png]
